# The wheat NB‐LRR gene *TaRCR1* is required for host defence response to the necrotrophic fungal pathogen *Rhizoctonia cerealis*

**DOI:** 10.1111/pbi.12665

**Published:** 2017-03-01

**Authors:** Xiuliang Zhu, Chungui Lu, Lipu Du, Xingguo Ye, Xin Liu, Anne Coules, Zengyan Zhang

**Affiliations:** ^1^ The National Key Facility for Crop Gene Resources and Genetic ImprovementInstitute of Crop ScienceChinese Academy of Agricultural SciencesBeijingChina; ^2^ School of Animal, Rural and Environmental SciencesNottingham Trent UniversityNottinghamUK

**Keywords:** NB‐LRR, *Rhizoctonia cerealis*, resistance, *Triticum aestivum*, common wheat, reactive oxygen species (ROS)

## Abstract

The necrotrophic fungus *Rhizoctonia cerealis* is the major pathogen causing sharp eyespot disease in wheat (*Triticum aestivum*). Nucleotide‐binding leucine‐rich repeat (NB‐LRR) proteins often mediate plant disease resistance to biotrophic pathogens. Little is known about the role of NB‐LRR genes involved in wheat response to *R. cerealis*. In this study, a wheat NB‐LRR gene, named *TaRCR1*, was identified in response to *R. cerealis* infection using Artificial Neural Network analysis based on comparative transcriptomics and its defence role was characterized. The transcriptional level of *TaRCR1* was enhanced after *R. cerealis* inoculation and associated with the resistance level of wheat. *TaRCR1* was located on wheat chromosome 3BS and encoded an NB‐LRR protein that was consisting of a coiled‐coil domain, an NB‐ARC domain and 13 imperfect leucine‐rich repeats. TaRCR1 was localized in both the cytoplasm and the nucleus. Silencing of *TaRCR1* impaired wheat resistance to *R. cerealis*, whereas *TaRCR1* overexpression significantly increased the resistance in transgenic wheat. TaRCR1 regulated certain reactive oxygen species (ROS)‐scavenging and production, and defence‐related genes, and peroxidase activity. Furthermore, H_2_O_2_ pretreatment for 12‐h elevated expression levels of *TaRCR1* and the above defence‐related genes, whereas treatment with a peroxidase inhibitor for 12 h reduced the resistance of *TaRCR1‐*overexpressing transgenic plants and expression levels of these defence‐related genes. Taken together, *TaRCR1* positively contributes to defence response to *R. cerealis* through maintaining ROS homoeostasis and regulating the expression of defence‐related genes.

## Introduction

Bread wheat (*Triticum aestivum*) is one of the most important staple crops. The wheat sharp eyespot disease, primarily caused by a necrotrophic fungal pathogen *Rhizoctonia cerealis*, is one of the destructive diseases of wheat in some regions of the world. In terms of wheat acreage affected by sharp eyespot, China is the largest epidemic region in the world, as exemplified by the 8.1 million hectares of wheat infected in 2005 (Chen *et al*., 2013) and 9.33 million hectares in 2015 (http://www.agri.cn/V20/bchqb/201501/t20150121_4344729.htm). Breeding to host resistance is an effective and environmentally friend way to protect wheat from *R. cerealis* infection. However, traditional resistance breeding is difficult as no wheat lines/cultivars with complete resistance to sharp eyespot have been identified, and the resistance in wheat accessions (CI12633, Luke and AQ24788‐83) is partial and controlled by multiple QTLs (quantitative trait loci, Cai *et al*., 2006; Chen *et al*., 2013). To improve wheat resistance to sharp eyespot, it is vital to identify genes that play important roles in the defence response and unravel their underlying functional mechanisms.

To control pathogens, plants activate defence mechanisms followed by pathogen detection through cell surface and intracellular immune receptors. Plants recognize pathogen‐associated molecular patterns (PAMPs) through cell‐surface pattern‐recognition receptors and sense pathogen effectors via intracellular nucleotide‐binding leucine‐rich repeat (NB‐LRR) proteins, resulting in PTI (PAMP‐triggered immunity) and ETI (effector‐triggered immunity), respectively (Jones and Dangl, 2006). Recently, to explain interactions between fungal pathogens and their host plants, Stotz *et al*. (2014) summarized another defence mechanism termed effector‐triggered defence (ETD). Compared with ETI, ETD responses against pathogens are relatively slow and not associated with a fast hypersensitive cell death response in hosts (Boys *et al*., 2012; Bozkurt *et al*., 2012; Jones and Dangl, 2006; Stotz *et al*., 2014; Thirugnanasambandam *et al*., 2011; Valent and Chang, 2010). Several dozens of *NB‐LRR* genes, acting as intracellular immune receptors to effectors of bacterial, viral and fungal pathogens, have been cloned from diverse plant species (Anderson *et al*., 1997; Cloutier *et al*., 2007; Deslandes *et al*., 1998; Ellis *et al*., 1999; Feuillet *et al*., 2003; Hinsch and Staskawicz, 1996; Huang *et al*., 2003, 2004; Inoue *et al*., 2013; Periyannan *et al*., 2013; Saintenac *et al*., 2013; Shen *et al*., 2007; Whitham *et al*., 1994). Recently, an emerging model is that NB‐LRR proteins often function in pairs, with ‘helper’ proteins required for the activity of ‘sensors’ that mediate pathogen recognition (Bonardi *et al*., 2011; Wu *et al*., 2016). Certain NB‐LRR proteins contribute to signal transduction and/or amplification (Bonardi *et al*., 2011; Césari *et al*., 2014; Gabriëls *et al*., 2007). The above‐mentioned NB‐LRRs play a pivotal role in plant resistance responses to biotrophic pathogens. However, in certain plant‐necrotrophic fungus pathosystems, the recognition of pathogen‐produced effectors by NB‐LRR proteins leads to effector‐triggered susceptibility (Faris *et al*., 2010; Lorang *et al*., 2007; Nagy and Bennetzen, 2008). For example, the wheat *Tns1* governs effector‐triggered susceptibility to two necrotrophic fungi *Stagonospora nodorum* and *Pyrenophora tritici‐repentis* (Faris *et al*., 2010). To our knowledge, no study about NB‐LRR genes involved positively in plant resistance responses to necrotrophic fungal pathogens has been reported yet.

Cellular redox status, including generation and scavenging of reactive oxygen species (ROS; including H_2_O_2_ and $O_{2}^{-}$), plays an important role in plant defence responses to pathogens. At the early stage of plant–pathogen interaction, oxidative burst is a common phenomenon coupled with the generation of ROS (Garcia‐Brugger *et al*., 2006). Necrotrophic pathogens also induce the generation of ROS (Foley *et al*., 2016; Heller and Tudzynski, 2011; Shetty *et al*., 2008). An appropriate level of ROS not only can promote cell wall reinforcement and phytoalexin production, but also has a signalling role in mounting a defence response (Quan *et al*., 2008). However, the overproduction of ROS may lead to oxidative stress that can damage some cellular compounds including proteins, lipids, carbohydrates and nucleotides of plant cells (Wu *et al*., 2008). ROS‐generating enzymes like NADPH‐dependent oxidase (NOX) complex, and various ROS‐scavenging systems, such as peroxidase, ascorbate peroxidase, catalase (CAT) and superoxide dismutase (SOD), are involved in fine‐tuning of ROS levels in the plant cells, resulting in the activation of plant defence responses (Mittler, 2002). However, knowledge about the involvement of ROS signalling in NB‐LRR‐mediated defence responses to fungal pathogens is limited.

An Artificial Neural Network (ANN) analysis on transcriptomic data has been used successfully to identify regulators of developmental processes in plants (Pan *et al*., 2013). In our laboratory, comparative transcriptomics based on microarray or RNA‐seq analysis have been used to identify genes expressed differentially between sharp eyespot‐resistant wheat CI12633 and susceptible wheat Wenmai 6 following infection with *R. cerealis*. In this study, ANN analysis of these transcriptomic data resulted in a regulatory network model of defence‐related genes, in which several potentially important genes (including the wheat NB‐LRR gene *TaRCR1*) in defence response to *R. cerealis* were predicted. The full‐length sequence of *TaRCR1* was isolated from the resistant wheat line CI12633. The defence role dissections via *TaRCR1*‐overexpression and *TaRCR1*‐silencing wheat plants indicated that *TaRCR1*, acting as a positive regulator, was required for host resistance response to *R. cerealis* infection through modulating the expression of several ROS‐scavenging and defence‐related genes.

## Results

### Identification of *TaRCR1* via transcriptomic and network inference analyses

**Figure 1:**
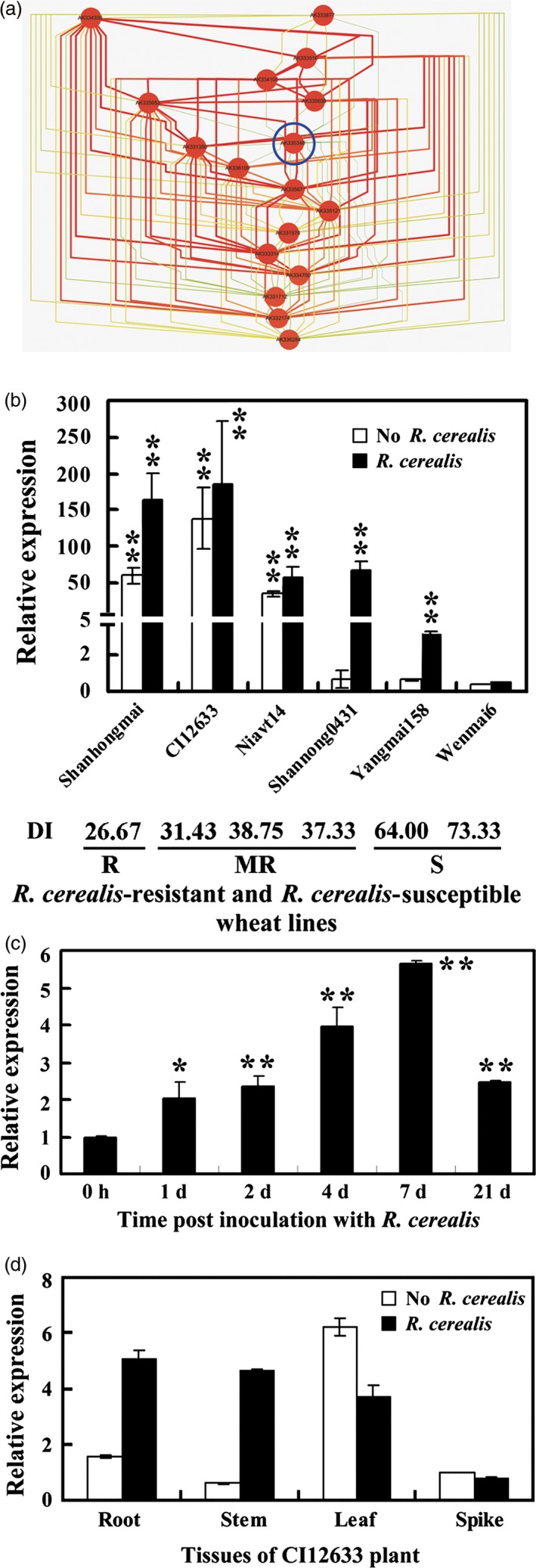
Transcription of *TaRCR1* in *Rhizoctonia cerealis*‐inoculated wheat (*Triticum aestivum*). (a) Regulatory interaction network of common top differentially expressed genes across the timecourse. Nodes were coloured based on the stress degree, red represented the highest stress, brown represented high stress degree, and yellow represented middle stress. The edge colour and thickness represent the degree of co‐expressed connections, from strong (thick and red colour) to weak (thin and green colour). The FoldChange of *TaRCR1* (AK335348) transcriptional level derived from microarray analysis (GEO accession number GSE69245) between the *R. cerealis*‐resistant wheat line CI12633/Shanhongmai and susceptible wheat cultivar Wenmai 6 at 4 and 21 days post inoculation (dpi) with *R. cerealis*. (b) Expression patterns of *TaRCR1* in six wheat cultivars with different degrees of resistance to *R. cerealis* R0301. The expression level of *TaRCR1* in the Wenmai 6 plants with mock treatment by sterile toothpicks without pathogen for 4 days was set to 1. R indicated resistant; MR indicated moderately resistant; S indicates susceptible; DI indicates disease index of sharp eyespot. (c) qRT‐PCR analysis of *TaRCR1* induction by *R. cerealis* R0301 inoculation in CI12633 plants. The expression level of *TaRCR1* at 0 dpi is set to 1. (d) Transcription of *TaRCR1* in roots, stems, leaves and spikes of CI12633 plants with inoculation by *R. cerealis* R0301 or mock treatment by sterile toothpicks without pathogen for 7 days. The transcriptional level of *TaRCR1* in spikes with mock treatment was set to 1. Statistically significant differences are derived from the results of three independent replications (*t*‐test: *, *P* < 0.05; **, *P* < 0.01).

Through microarray analysis, we identified 1533 genes, which were expressed in more than twofold higher level in *R. cerealis*‐resistant line CI12633/Shanhongmai than in *R. cerealis*‐susceptible cultivar Wenmai 6 following 4 and 21 day postinoculation (dpi) with *R. cerealis* isolate R0301 (GEO accession number GSE69245). ANN analysis on the microarray data resulted in a regulatory network model of defence‐related genes and predicted several genes with major highly interacting nodes, including one key gene *TaRCR1* (NCBI accession no. AK335348 harbouring the probe sequence with TIGR number TC376099) (Fig. 1a). The transcriptional levels of the probe (TIGR number: TC376099), corresponding to the 3ʹ terminal sequence of *TaRCR1*, were significantly higher in these two‐resistant wheat lines CI12633 (about 341‐fold at 4 dpi with *R. cerealis* R0301 and 409‐fold at 21 dpi) and Shanhongmai (about 162‐fold at 4 dpi and 319‐fold at 21 dpi) than in the susceptible wheat Wenmai 6 (Figure S1). Without or with *R. cerealis* inoculation for 4 day, the transcriptional levels of *TaRCR1* in six wheat lines/cultivars with different resistance degrees, including sharp eyespot‐resistant lines CI12633 and Shanhongmai, moderately resistant lines Niavt 14 and Shannong 0431, moderately susceptible wheat cultivar (cv.) Yangmai 158 and susceptible cv. Wenmai 6 were investigated (Figure 1b). Either with or without *R. cerealis* infection, the expression level of *TaRCR1* was the highest in CI12633, slightly decreased in Shanhongmai, gradually declined in Niavt 14 and Shannong 0431, and reached the lowest in Wenmai 6 (Figure 1b). The results suggested that the transcriptional level of *TaRCR1* was associated with wheat resistance degrees to *R. cerealis*. Furthermore, *TaRCR1* transcription in CI12633 stems was enhanced by *R. cerealis*, and the induction reached a peak at 7 dpi with *R. cerealis* (Figure 1c). Additionally, the expression analyses of *TaRCR1* in organs of CI12633 plants showed that, under control treatment with sterile toothpick without *R. cerealis*,* TaRCR1* was expressed in higher levels in the leaves than in the other organs; after 7 dpi with *R. cerealis*, levels in the roots and stems, the main disease‐occurring sites, were more abundant than those in the leaves and spikes (Figure 1d). These results suggested that *TaRCR1* may participate in the wheat defence response to *R. cerealis*.

### Sequence characterization of *TaRCR1* in wheat

**Figure 2:**
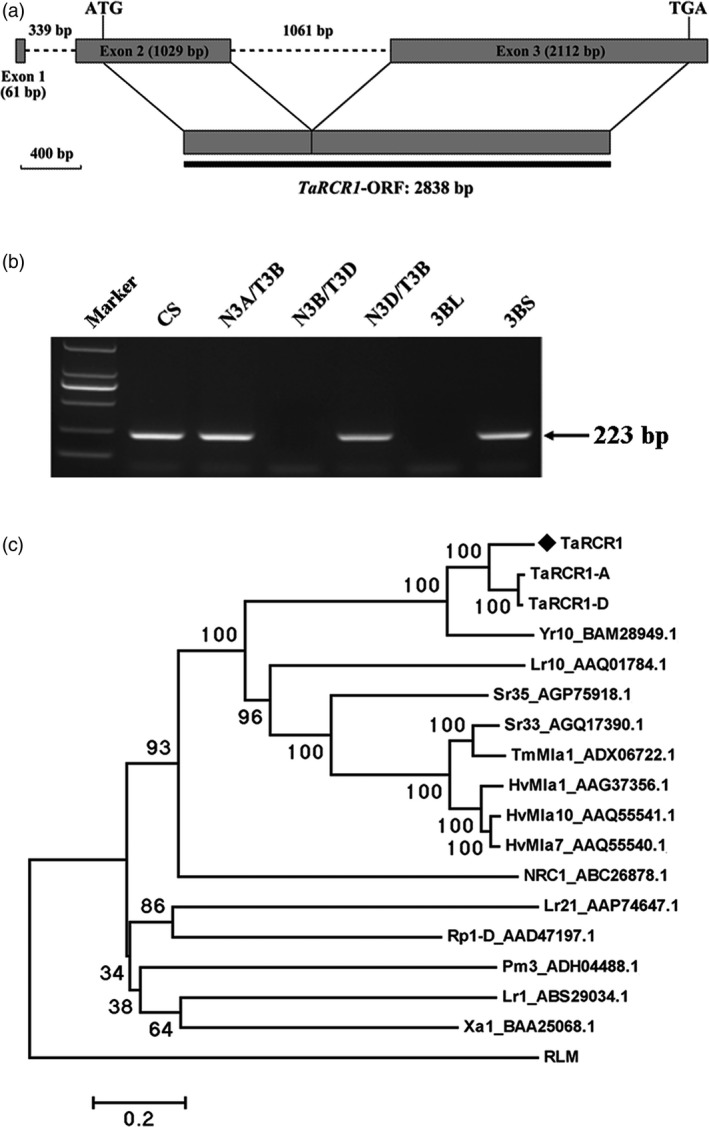
Gene structure, chromosome location and phylogenetic tree analysis of *TaRCR1*. (a) Genomic structure of *TaRCR1* gene; dark grey portions and dotted lines represent exons and introns, respectively. The dark line is the complete open reading frame. (b) Chromosome location of *TaRCR1* using nulli‐tetrasomic and double ditelosomic lines derived from wheat cv. Chinese Spring (CS). Marker, DL2, 000 DNA marker; N3A/T3B, N3B/T3D, N3D/T3B, three nulli‐tetrasomic lines; 3BL and 3BS, two di‐telosomic lines of chromosome 3B. (c) Phylogenetic analysis of the deduced amino acid sequences of TaRCR1 and other plant NB‐LRR proteins. Yr10 and Xa1, *Oryza sativa* Yr10 and Xa1; Lr1, Lr10, and Sr33, *Triticum aestivum* Lr1, Lr10, and Sr33; Sr35 and TmMla1, *Triticum monococcum* Sr35 and TmMla1; HvMla1, HvMla7, and HvMla10, *Hordeum vulgare* HvMla1, HvMla7, and HvMla10; NRC1, *Solanum lycopersicum* NRC1; Rp1‐D, *Zea mays* Rp1‐D; RLM, *Arabidopsis thaliana* RLM. The black diamond indicates the position of TaRCR1.

The full‐length cDNA and genomic sequences of *TaRCR1* were cloned from CI12633. The comparison of the cDNA and genomic sequences showed that *TaRCR1* genomic sequence with 4602‐bp length was comprised of two introns and three exons. The *TaRCR1* mRNA (GenBank accession no. KU161103) contains an open reading frame (ORF) with 2838‐bp length, the 5′‐untranslated region (UTR) with 235 bp and 3′‐UTR with 129 bp (Figure 2a). The deduced protein TaRCR1 consisted of 945 amino acid (AA) residues with a molecular weight of 106.28 kD and a theoretical *iso*‐electric point (pI) of 8.65. Analysis of the protein sequence showed that TaRCR1 was a typical NB‐LRR and contained an N‐terminal coiled‐coil (CC) domain (AAs 1‐180), an NB‐ARC domain (AAs 181‐567) and 13 imperfect LRRs (AAs 568‐945) at the C‐terminus. A conserved EDVID motif (EDCID in TaRCR1, AAs 80‐84) was identified in the CC domain of TaRCR1 (Figure S2). All of the important motifs, including P‐loop, RNBS‐A, Walker B, RNBS‐B, GLPL, RNBS‐D and MHD, present in the NB‐ARC domain characteristic of typical NB‐LRR proteins (Ellis *et al*., 2000), were found in TaRCR1 protein (Figure S2). Although the full length of *TaRCR1* was not obtained from *R. cerealis*‐susceptible wheat, the 3ʹ‐terminal sequence of *TaRCR1* was obtained from *R. cerealis*‐susceptible cultivar Wenmai 6. Comparison of the 3ʹ‐terminal sequences of *TaRCR1* from CI12633 and Wenmai 6 showed that shared 60.44% identity and many single nucleotide polymorphisms (SNPs) existed at their 3ʹ‐terminal sequences (Figure S3).

Sequence alignment of *TaRCR1* with the draft sequences of hexaploid bread wheat chromosomes from the International Wheat Genome Sequencing Consortium (IWGSC, http://www.wheatgenome.org/) suggested that *TaRCR1* should be located on wheat chromosome 3B. *TaRCR1* gene‐specific PCR amplification on the templates from the genomic DNAs of wheat Chinese Spring (CS) nulli‐tetrasomic and di‐telosomic lines further indicated that *TaRCR1* was located on the short arm of wheat chromosome 3B (Fig. 2b). The ORF sequences of *TaRCR1‐A* (GenBank accession no. KX840356) and *TaRCR1‐D* (GenBank accession no. KX840357) located on chromosome 3AS and 3DS were cloned from CI12633, respectively. Both homoeologous proteins consisted of 955 AAs. TaRCR1, its homologs and some NB‐LRR proteins with known resistance function were subjected to identity and phylogenetic tree analyses. The results indicated that the TaRCR1 protein sequence shared 82.45%, 81.38% and 61.28% identities with TaRCR1‐A, TaRCR1‐D and rice Yr10 (GenBank accession no. BAM28949.1), respectively, and they were clustered on the same branch (Figure 2c). However, the TaRCR1 protein shared quite low identities (7.05%–26.12%) with other plant NB‐LRRs (Figure 2c; Table S1). These results suggested that TaRCR1 was a member of the NB‐LRR family in wheat.

### TaRCR1 localizes in both the cytoplasm and the nucleus

**Figure 3:**
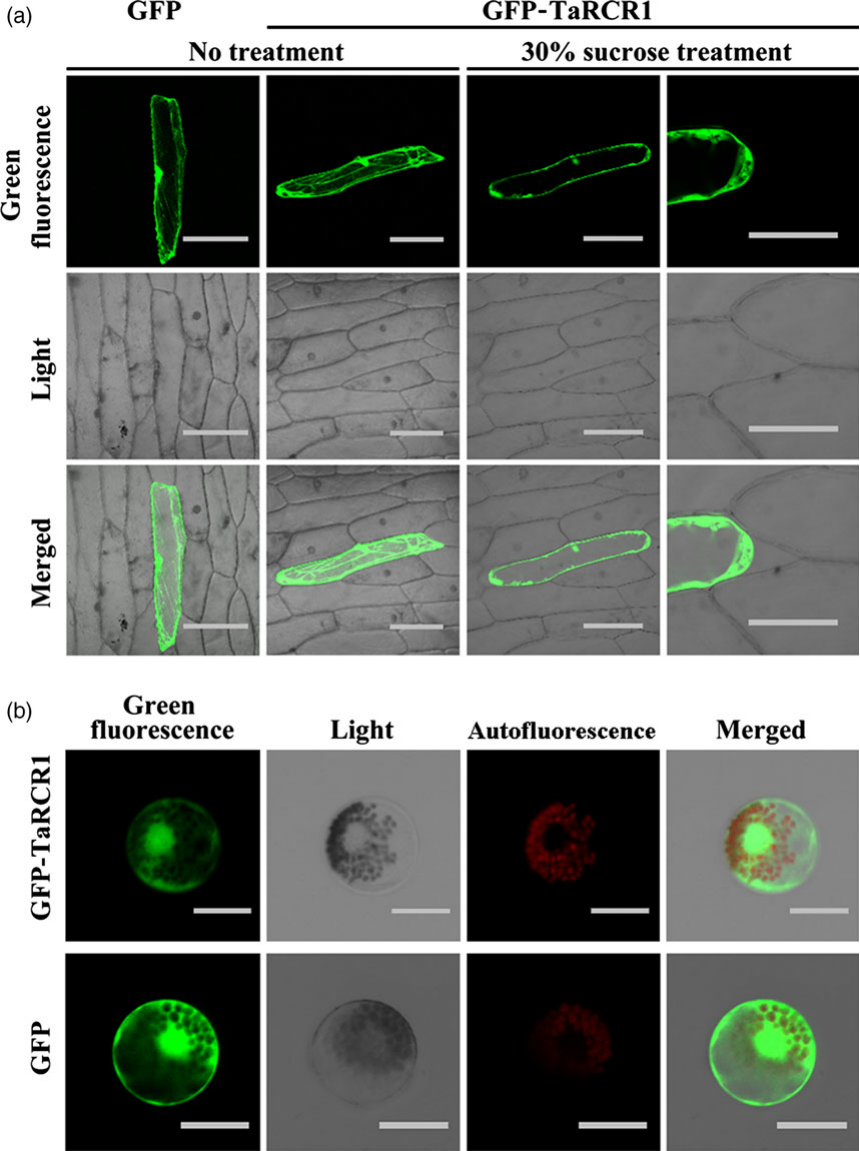
Subcellular localization of the green fluorescent protein (GFP)‐TaRCR1 fusion protein in onion epidermal cells and wheat protoplasts. (a) The control GFP and fused GFP‐TaRCR1 in onion epidermal cells. After transformed with GFP‐TaRCR1 for 20 h, the onion epidermal cells were plasmolysed by 30% sucrose treatment for 10 min, and then images were taken using a confocal microscope with 536 nm wavelengths. Bars = 100 μm. (b) The control GFP and fused GFP‐TaRCR1 in wheat protoplasts. The autofluorescence was from wheat chloroplast. Confocal images were taken at 20 h after transformation using 536 nm wavelengths. Bars = 20 μm.

To investigate the subcellular localization of TaRCR1 in the plant cells, the p35S:GFP‐TaRCR1 fusion vector was constructed, and the p35S:GFP‐TaRCR1 and the control p35S:GFP vectors were separately introduced into and transiently expressed in either onion epidermal cells or wheat mesophyll protoplasts. Confocal microscopic examination showed that in the transformed onion epidermal cells, the GFP‐TaRCR1 protein was expressed and localized in both the cytoplasm and nucleus (Figure 3a). After plasmolysis of the onion epidermal cells, TaRCR1 was more clearly observed in both the cytoplasm and nucleus (Figure 3a). Moreover, in the wheat mesophyll protoplasts, the GFP‐TaRCR1 protein distributed in both the cytoplasm and nucleus (Figure 3b), the red colour was auto‐fluorescence from wheat chloroplast (Figure 3b). The control p35S:GFP protein diffused in both the nucleus and cytoplasm (Figure 3a, b). These results indicated that TaRCR1 localizes in both the cytoplasm and nucleus in wheat.

### Silencing of *TaRCR1* impairs host resistance to *R. cerealis*

**Figure 4:**
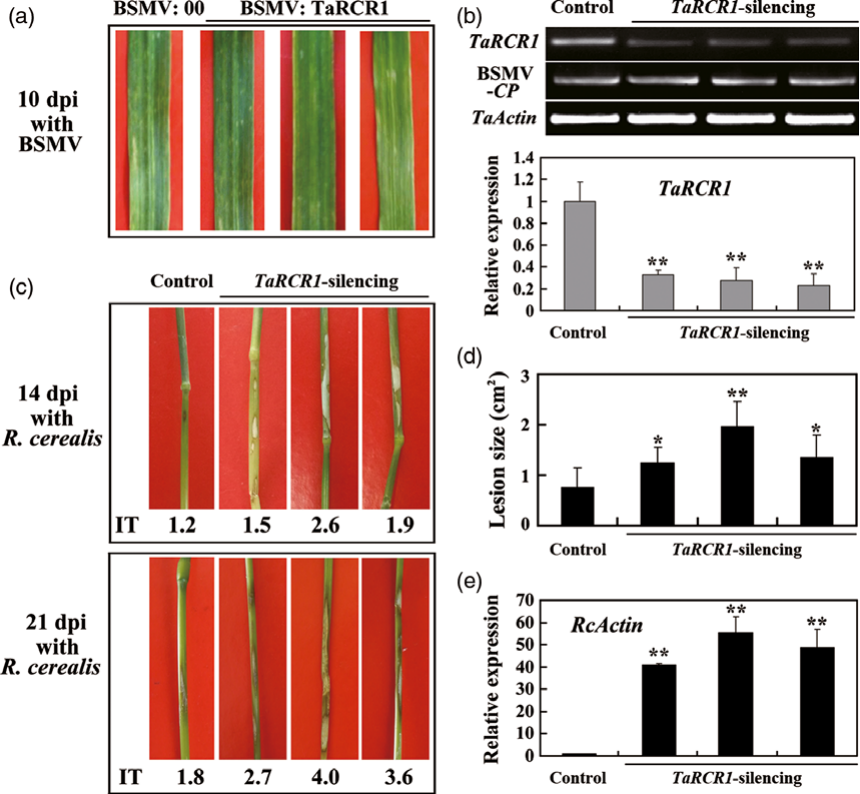
Silencing of *TaRCR1* by barley stripe mosaic virus (BSMV)‐induced gene silencing impairs CI12633 resistance to *Rhizoctonia cerealis* WK207. (a) Mild chlorotic mosaic symptoms were observed on leaves at 10 days postinoculated (dpi) with BSMV:00 or BSMV:TaRCR1. (b) (q)RT‐PCR analyses of the transcription levels of BSMV coat protein (*CP*) and wheat *TaRCR1* genes in the wheat plants infected by BSMV:00 or BSMV:TaRCR1 at 14 dpi with *R. cerealis*. The relative transcript level of *TaRCR1* in BSMV:TaRCR1‐infected (*TaRCR1*‐silencing) wheat CI12633 plants is relative to that in BSMV:00‐infected (control) plants (set to 1). (c) Sharp eyespot symptoms of the control and *TaRCR1*‐silencing CI12633 plants at 14 and 21 dpi with *R. cerealis*. (d) Disease lesion size in *TaRCR1*‐silencing and control CI12633 plants at 21 dpi with *R. cerealis*. (e) qRT‐PCR analysis of *R. cerealis actin* (*RcActin*) gene in stems of *TaRCR1*‐silencing and control CI12633 plants represented the biomass of *R. cerealis*. Significant differences were analyzed based on three replications (*t*‐test: *, *P* < 0.05; **, *P* < 0.01). Error bars indicate SE.

To explore whether *TaRCR1* was required for wheat resistance to *R. cerealis*, barley stripe mosaic virus (BSMV)‐based virus‐induced gene silencing (VIGS) was used to knockdown *TaRCR1* transcript in the partially resistant line CI12633. A 3ʹ‐terminal fragment specific to *TaRCR1* was inserted in an antisense orientation into *Nhe* I restriction site of the BSMV RNAγ chain to generate BSMV:TaRCR1 recombinant construct (Figure S4). Following inoculation with BSMV:TaRCR1 or BSMV:00 (as a control) viruses for 10 day, the mild chlorotic mosaic symptoms of BSMV appeared in the leaves of the infected CI12633 plants (Figure 4a), and the expression of BSMV coat protein (*CP*) gene was detected (Figure 4b), proving that these inoculated plants were successfully infected by BSMV. The BSMV:00 infection did not significantly affect the expression of *TaRCR1* in CI12633 plants (Figure S5), whereas the transcript level of *TaRCR1* was markedly reduced in BSMV:TaRCR1‐infected (*TaRCR1*‐silencing) plants (Figure 4b). Subsequently, *R. cerealis* WK207 was inoculated on the stems of these BSMV‐infected plants to evaluate the defence role of *TaRCR1*. At 14 dpi with *R. cerealis*, lesions (symptom of sharp eyespot disease) were present on the stems of *TaRCR1*‐silencing plants, but to a lesser extent in BSMV:00‐treated control plants (Figure 4c). At 21 dpi with *R. cerealis*, the lesion areas on the stems of *TaRCR1*‐silencing plants were 1.24–1.96 cm^2^, while the average lesion area of BSMV:00‐treated plants was only 0.75 cm^2^ (Figure 4d); the infection types (ITs) of *TaRCR1*‐silencing plants were ranged from 2.7 to 4.0, whereas the average IT of BSMV:00‐treated plants was 1.8 (Figure 4c). Relative abundances of *R. cerealis actin* mRNA, as a measure of the fungal growth in the pathogen‐inoculated wheat, were consistent with the symptoms of these plants (Figure 4e). These results indicated that the silence of *TaRCR1* in CI12633 plants impaired resistance to *R. cerealis* and that *TaRCR1* is required for the wheat defence response to *R. cerealis* infection.

### *TaRCR1* overexpression improves resistance to *R. cerealis* in transgenic wheat

**Figure 5:**
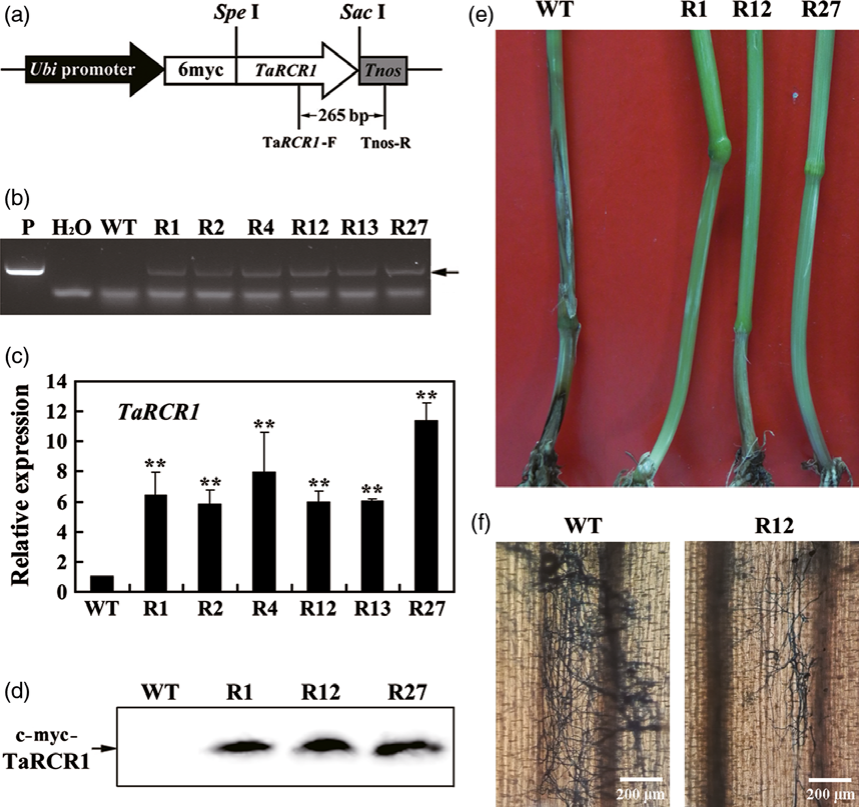
Molecular characterizations of *TaRCR1*‐overexpressing wheat plants and responses to *Rhizoctonia cerealis* infection. (a) *TaRCR1‐*overexpressing transformation vector pUbi:myc‐TaRCR1. The arrow indicates the fragment amplified in the PCR detection of the transgene. (b) PCR pattern of *TaRCR1*‐overexpressing transgenic lines and wild‐type (WT) wheat Yangmai 16 using the primers specific to *TaRCR1‐Tnos* cassette. P, the transformation vector pUbi: myc‐TaRCR1 as a positive control. (c) qRT‐PCR analyses of the relative transcript levels of *TaRCR1* in *TaRCR1* transgenic lines at 7 day postinoculation (dpi) with *R. cerealis*. Three biological replicates per line were averaged and statistically treated (*t*‐test; **P < 0.01). Bars indicate standard error of the mean. (d) Western blot pattern of the three *TaRCR1*‐overexpressing transgenic lines and WT Yangmai 16 using an anti‐myc antibody. Similar results were obtained from three independent replicates. (e) Typical symptoms of sharp eyespot in the three *TaRCR1*‐overexpressing transgenic and WT Yangmai 16 plants. IT indicates infection type. (f) Trypan blue staining for the detection of the *R. cerealis* hyphae on the base leaf sheath of the *TaRCR1* transgenic and WT Yangmai 16 plants at 14 dpi with *R. cerealis* WK207.

To further investigate the role of *TaRCR1* in the defence against *R. cerealis*, the overexpression transformation construct pUbi:myc‐TaRCR1 (Figure 5a) was generated and transformed into moderately susceptible wheat cultivar Yangmai 16. PCR analysis using the primers specific to the c‐myc‐TaRCR1‐Tnos chimera showed that the introduced transgene could be detected in six *TaRCR1*‐overexpressing lines (R1, R2, R4, R12, R13 and R27) from T_0_ to T_4_ generations (Figure 5b). qRT‐PCR results showed that the transcript abundances of *TaRCR1* in these six transgenic lines were markedly elevated compared with wild‐type (WT) Yangmai 16 (Figure 5c). Western blotting indicated that the introduced *c‐myc‐TaRCR1* gene could be expression in the fusion protein in these *TaRCR1‐*overexpressing transgenic lines, but not in WT Yangmai 16 (Figure 5d). Following inoculation with *R. cerealis* isolates R0301 (T_1_–T_2_ plants) and WK207 (T_3_–T_4_ plants), compared with WT Yangmai 16, these *TaRCR1*‐overexpressing lines exhibited significantly enhanced resistance to *R. cerealis* (Figure 5e; Table 1). The average disease indexes of these *TaRCR1*‐overexpressing lines in T_1_–T_4_ generations infected with *R. cerealis* were ranged from 24.75 to 45.00, whereas those of WT Yangmai 16 were 50.82–57.11 (Table 1). Furthermore, microscopic observation indicated that the hyphae abundances of *R. cerealis* strain WK207 were less on the inoculated base sheaths of the *TaRCR1*‐overexpressing line R27 than on those of WT Yangmai 16 (Figure 5f), providing supporting evidence that *TaRCR1* overexpression increases resistance to hyphae development of *R. cerealis*. These results indicated that TaRCR1 positively regulates wheat resistance response to sharp eyespot caused by *R. cerealis*.

**Table 1:** *Rhizoctonia cerealis* responses of transgenic wheat (*Triticum aestivum*) lines overexpressing *TaRCR1* and wild‐type wheat lines†

Lines	Infection type				Disease index			
	T_1_	T_2_	T_3_	T_4_	T_1_	T_2_	T_3_	T_4_
R1	1.52**	2.25*	1.83*	1.51**	30.33**	45.00*	36.67*	30.33**
R2	2.10*	1.32**	1.75**	2.25*	42.05*	26.43**	35.00**	45.00*
R4	1.72**	1.72**	1.67**	1.83*	34.47**	34.39**	33.33**	36.61*
R12	1.28**	1.36**	2.17*	2.17*	25.50**	27.27**	43.33*	43.33*
R13	1.58**	1.54**	1.63**	1.81*	31.66**	30.86**	32.50**	36.25*
R27	1.80*	1.24**	1.71**	1.63**	36.06*	24.75**	34.29**	33.28**
WT	2.86	2.70	2.63	2.54	57.11	53.90	52.63	50.82

* or ** significant difference between each transgenic line and WT wheat at *P* < 0.05 or 0.01 (*t*‐test). ^†^R1, R2, R4, R12, R13, R27 indicate *TaRCR1*‐overexpressing wheat lines; WT indicates untransformed wild‐type Yangmai 16*. Rcerealis* isolate R0301 was used to infect plants in T_1_ and T_2_ generations, and *R. cerealis* isolate WK207 was used to infect plants in T_3_ and T_4_ generations. At least 10 plants for each line were assessed for disease intensity.

### TaRCR1 modulates expression levels of defence‐associated genes

**Figure 6:**
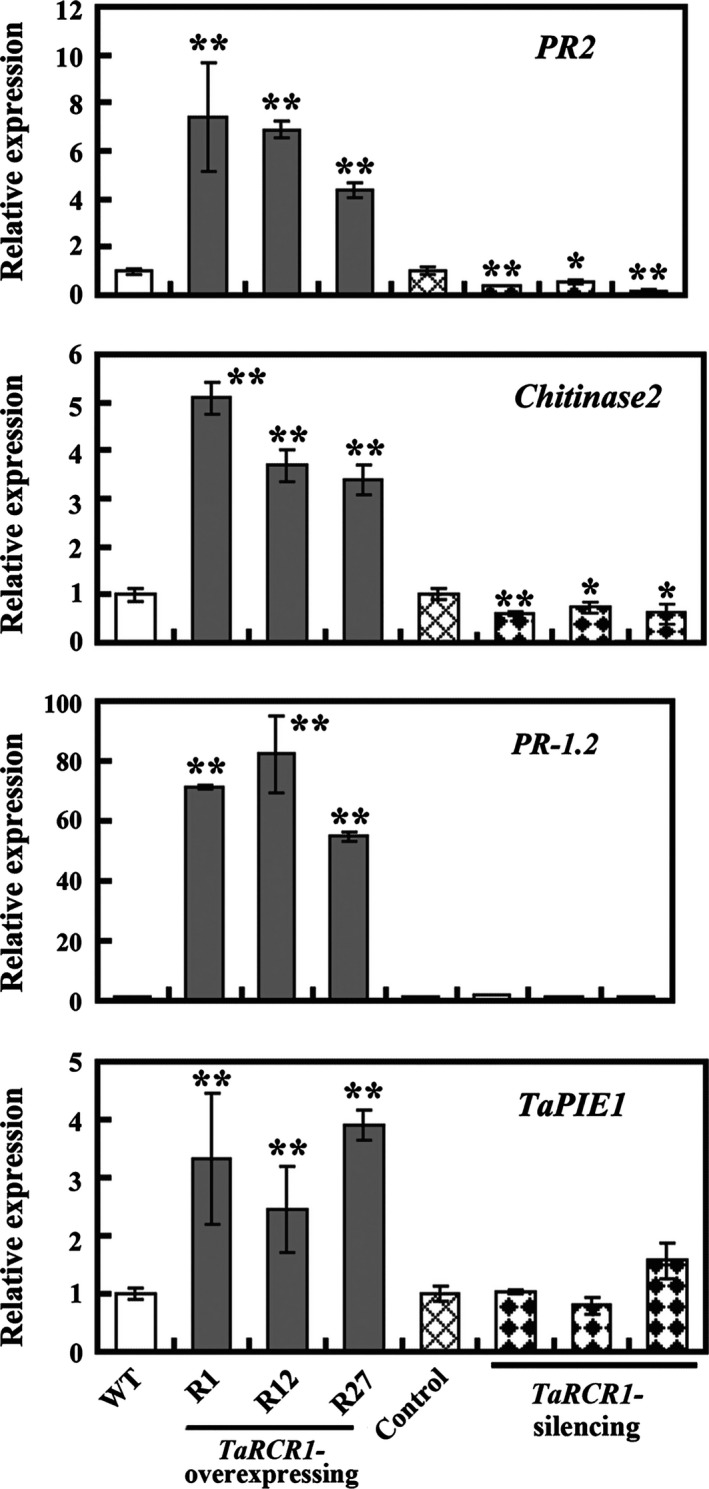
Transcription analyses of four genes (*PR2*,* chitinase 2*,*PR‐1.2* and *TaPIE1*) related to disease resistance in wheat (*Triticum aestivum*) after *Rhizoctonia cerealis* infection. The reported transcript levels of the tested genes in the *TaRCR1*‐overexpressing (R1, R12, R27) and *TaRCR1*‐silencing plants are relative to those in the wild‐type (WT) Yangmai 16 and BSMV:00‐infected control plants, respectively. Statistically significant differences were analyzed based on three technical replications (*t*‐test; *P <* *0.05, **P <* *0.01). Bars indicate standard error of the mean.

Seven wheat defence‐associated genes, including *TaPIE1* (GenBank accession no. EF583940), *defensin* (GenBank accession no. CA630387), *PR‐1.2* (GenBank accession no. AJ007349), *PR2* (GenBank accession no. AF112965), *PR10* (GenBank accession no. CA613496), *chitinase1* (GenBank accession no. CK207575) and *chitinase2* (GenBank accession no. TC426538), have been implicated in resistance responses to *R. cerealis* (Chen *et al*., 2008; Wei *et al*., 2016; Zhu *et al*., 2014, 2015). Additionally, based on the microarray data (GEO accession number GSE69245), *chitinase2* and *TaPIE1* were expressed in higher levels in CI12633 and Shanhongmai than in Wenmai6 (Table S2). To examine whether TaRCR1 regulates defence‐associated genes in wheat response to *R. cerealis*, we have analyzed expression patterns of the above‐mentioned seven defence‐associated genes in *TaRCR1*‐overexpressing and *TaRCR1*‐silencing wheat plants as well as the control plants. The results showed that following inoculation with *R. cerealis* WK207 for 7 day, the transcriptional levels of *PR2* and *chitinase2* were significantly decreased in *TaRCR1*‐silencing plants compared with BSMV:00‐infected control plants, whereas they were significantly increased in *TaRCR1*‐overexpressing lines compared with WT Yangmai 16 (Figure 6). The transcriptional levels of *PR‐1.2* and *TaPIE1* were higher in *TaRCR1*‐overexpressing lines than in WT Yangmai 16 plants (Figure 6). Additionally, the transcriptional levels of *PR2*,* chitinase2*,* PR‐1.2* and *TaPIE1* were significantly induced in WT Yangmai 16 and more markedly induced in *TaRCR1‐*overexpressing wheat lines than the WT after *R. cerealis* WK207 inoculation for 7 day (Figure S6).

### ROS homoeostasis is crucial for *TaRCR1*‐mediated resistance against *R. cerealis*

**Figure 7:**
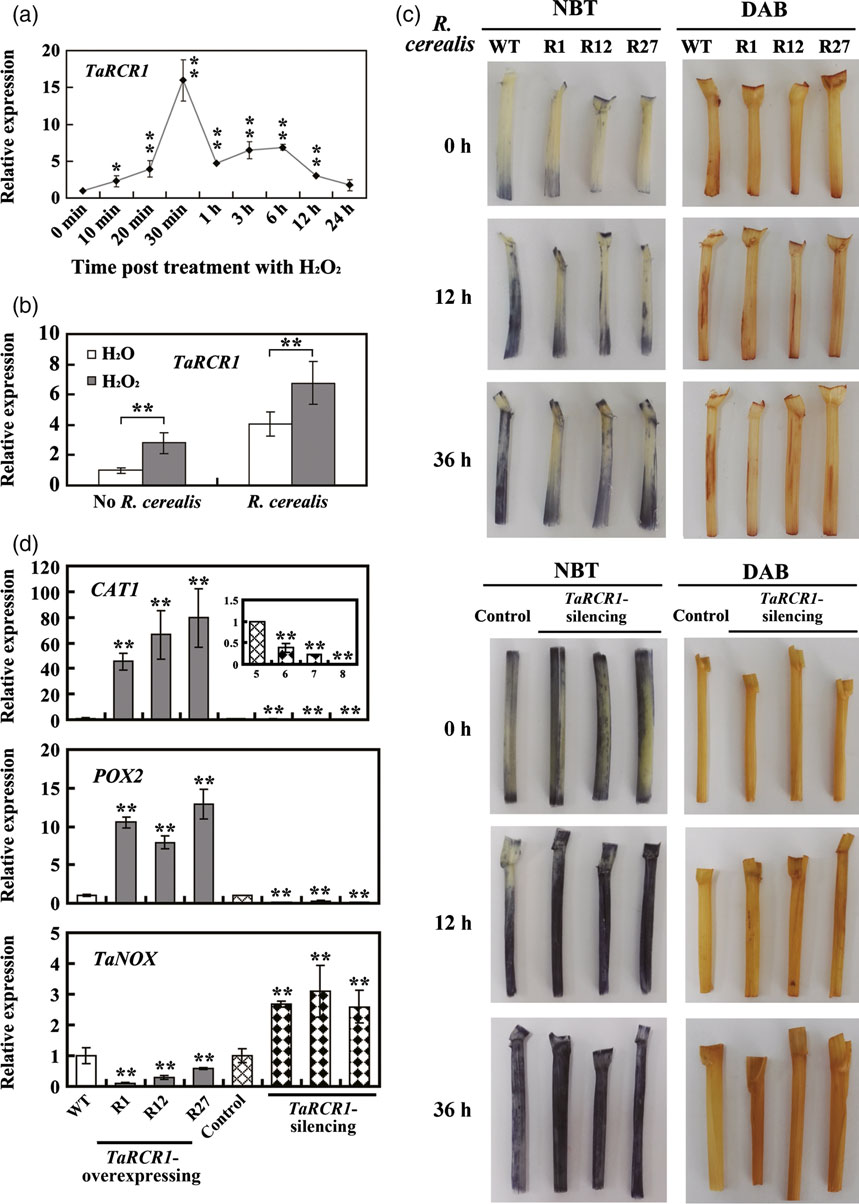
TaRCR1‐mediated resistance to *Rhizoctonia cerealis* is associated with ROS homoeostasis. (a) Expression of *TaRCR1* in leaves of Yangmai 16 wheat in response to exogenous applications of H_2_O_2_. Wheat plants at four‐leaf stage were sprayed with 10 mM H_2_O_2_ and 0.1% Tween‐20 (as a control). (b) Transcript induction of *TaRCR1* by *R. cerealis* is regulated by H_2_O_2_ treatment. Total RNA was extracted from the sheaths of Yangmai 16 plants at four‐leaf stage pretreated with H_2_O or H_2_O_2_ for 12 h, followed by *R. cerealis* treatment for 4 day. (c) Detection of hydrogen peroxide (H_2_O_2_) and superoxide anion ($O_{2}^{-}$) in wheat. Sheaths were harvested from wheat plants at 0, 12 and 36 hpi with *R. cerealis* and were then stained with 3,3ʹ‐diaminobenzidine (DAB) for H_2_O_2_ detection and nitroblue tetrazolium (NBT) for $O_{2}^{-}$ detection, respectively. Similar results were obtained from three independent replicates. WT: untransformed wheat Yangmai 16. R1, R12, R27: *TaRCR1*‐overexpressing lines. (d) Transcriptional analysis of genes encoding ROS‐scavenging enzymes (CAT1 and POX2) and ROS‐producing enzyme NOX in wheat sheaths. Statistically significant differences of *TaRCR1*‐overexpressing (R1, R12, R27) or *TaRCR1*‐silencing wheat plants were compared with the WT or the BSMV:00‐infected control based on three technical replications (*t*‐test; *P <* *0.05, **P <* *0.01). Bars indicate standard error of the mean.

To explore whether *TaRCR1*‐mediated resistance against *R. cerealis* is closely linked to the homoeostasis between ROS scavenging and production, we performed the following experiments. After H_2_O_2_ treatment, the expression level of *TaRCR1* was dramatically induced from 10 min to 24 h, which reached the first peak at 30 min (16.20‐fold); and the second peak at 3 h (7.30‐fold) (Figure 7a). Interestingly, H_2_O_2_ pretreatment elevated the transcriptional induction level of *TaRCR1* by *R. cerealis* WK207 inoculation (Figure 7b). The 3,3ʹ‐diaminobenzidine (DAB) and nitroblue tetrazolium (NBT) stains were used to detect H_2_O_2_ and $O_{2}^{-}$ in wheat sheaths. Without *R. cerealis* inoculation, little H_2_O_2_ and O_2_^‐^ were detected (Figure 7c). After *R. cerealis* inoculation for 12 and 36 h, accumulation of H_2_O_2_ and $O_{2}^{-}$ was more in *TaRCR1*‐silencing plants than that in control plants, but was the lowest in *TaRCR1*‐overexpressing plants (Figure 7c). At 4 dpi with *R. cerealis*, the accumulation of H_2_O_2_ and $O_{2}^{-}$ in all infected sheaths declined (Figure S7). These results implied the ROS homoeostasis is associated with *TaRCR1*‐mediated resistance against *R. cerealis*.

Following *R. cerealis* WK207 inoculation for 7 day, the transcriptional levels of two wheat ROS‐scavenging enzyme genes, including a CAT gene *CAT1* (GenBank accession no. AJ007349) and a peroxidase gene *POX2* (GenBank accession no. X85228), were significantly lower in *TaRCR1*‐silencing plants than in the control plants, but were the highest in *TaRCR1*‐overexpressing plants. However, the transcript level of a ROS‐producing enzyme gene *NOX* (GenBank accession no. AY561153) was significantly elevated in *TaRCR1*‐silencing plants than in the control plants, but the lowest in *TaRCR1*‐overexpressing plants (Figure 7d). These results suggested that TaRCR1 maintained the ROS homoeostasis possibly through regulating positively the transcription of *CAT1* and *POX2* genes but negatively the expression of *NOX*.

**Figure 8:**
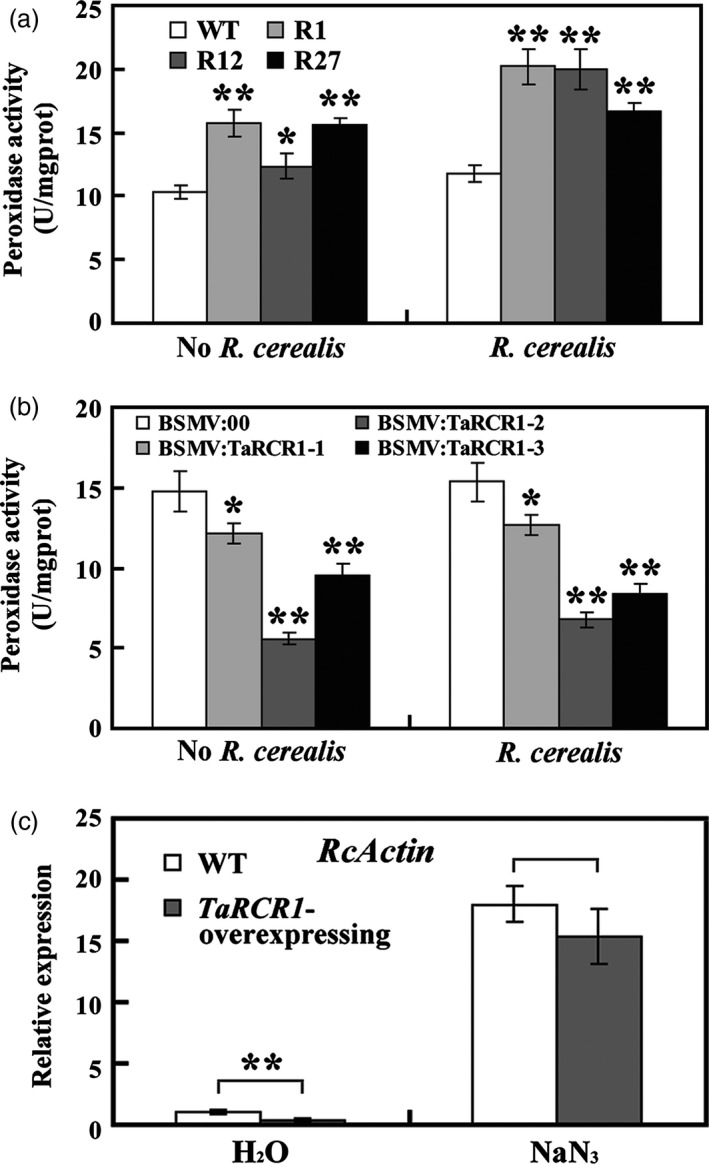
Analysis of peroxidase activity and effect of NaN_3_ treatment on TaRCR1‐mediated resistance to *Rhizoctonia cerealis*. (a, b) Analysis of peroxidase activity in *TaRCR1*‐overexpressing lines (R1, R12, R27), WT,* TaRCR1*‐silencing plants (BSMV:TaRCR1‐1, BSMV:TaRCR1‐2 and BSMV:TaRCR1‐3) and BSMV:00‐infected controls before and after *R. cerealis* inoculation for 12 h. Statistically significant differences of *TaRCR1*‐overexpressing or *TaRCR1*‐silencing wheat plants were compared with the WT or the control based on three independent replications (*t*‐test; *P <* *0.05, **P <* *0.01). mgprot, mg protein. (c) qRT‐PCR analysis of *R. cerealis actin* (*RcActin*) gene represented the biomass of *R. cerealis* in *TaRCR1*‐overexpressing and WT plants after pretreatments with H_2_O and NaN_3_ (peroxidase biosynthesis inhibitor) for 12 h then *R. cerealis* inoculation for 7 day.

Before *R. cerealis* inoculation, peroxidase activity was the highest in *TaRCR1*‐overexpressing plants, the lowest in *TaRCR1*‐silencing plants (Figure 8a, b). After *R. cerealis* WK207 inoculation for 12 h, the increased activity of peroxidase was still the highest in the transgenic wheat plants and the lowest in *TaRCR1*‐silencing wheat plants (Figure 8a, b). To further address the relationship between the TaRCR1‐mediated resistance and ROS homoeostasis, *TaRCR1*‐overexpressing and the WT plants were pretreated with NaN_3_, a potential inhibitor that has been reported to suppress peroxidase (Zhan *et al*., 2003), and then inoculated with *R. cerealis* WK207. As shown in Figure 8c, after pretreatment with water (containing 0.1% Tween‐20) for 12 h, the relative biomass of *R. cereal* was significantly less in *TaRCR1*‐overexpressing plants (0.36‐fold) than in the WT plants, further indicating that *TaRCR1* overexpression enhanced resistance to the pathogen. After NaN_3_ pretreatment for 12 h, the relative biomass of *R. cerealis* in *TaRCR1*‐overexpressing plants was remarkably increased and was only slightly lower (0.85‐fold) than in WT plants (Figure 8c). These results suggested that the inhibition of peroxidase impaired TaRCR1‐mediated resistance to *R. cerealis* and that ROS homoeostasis is important for the TaRCR1‐mediated resistance.

Furthermore, following H_2_O_2_ treatment for 12 h, the transcriptional levels of *PR2*,* chitinase2*,* PR‐1.2* and *TaPIE1* were significantly elevated (Figure S8), suggesting that these defence‐related genes were responsive to H_2_O_2_ stimuli. NaN_3_ treatment decreased the transcriptional levels of *PR2*,* chitinase2* and *TaPIE1* (Figure S8), suggesting that these three genes were regulated by peroxidase‐mediated ROS scavenging.

## Discussion

In this study, a wheat NB‐LRR gene, namely *TaRCR1*, was identified and cloned based on comparative transcriptomics. Microarray data showed that *TaRCR1* processed significantly higher transcriptional levels in *R. cerealis*‐resistant wheat lines CI12633 and Shanhongmai than in the susceptible wheat Wenmai 6. Additionally, the RNA‐Seq analysis (data not shown) showed that the transcriptional level of *TaRCR1* was markedly higher (76.01‐fold) in CI12633 than in Wenmai 6. Usually, the change in expression of *R* genes (encoding NB‐LRR immune receptors) is small and these *R* genes could not be picked up by microarray or other transcriptomic analyses. Accumulating evidence indicates that plant NB‐LRR proteins not only act as immune receptors, but also contribute to signal transduction and/or amplification in plant–pathogens (Wu *et al*., 2016). For example, the tomato NRC1, which was identified by combination techniques of cDNA‐amplified fragment length polymorphism and VIGS, functioned in cell death signalling pathways and contributed to resistance response to *Cladosporium fulvum* (Gabriëls *et al*., 2007). We thus deduced that TaRCR1 might participate in defence signalling transduction and/or positive regulator. The qRT‐PCR analysis showed that the transcriptional level of *TaRCR1* was associated with the resistance degree in six different wheat lines/cultivars. The 3ʹ‐terminal sequences of *TaRCR1* from the *R. cerealis*‐resistant wheat line CI12633 and the homologous sequence from *R. cerealis*‐susceptible wheat cv. Wenmai 6 shared 60.44% identity and many SNPs existed at their 3ʹ‐terminal sequences, resulting in some nonsynonymous mutations and the transcript stability (Spies *et al*., 2013), which might be one reason for the distinct transcript abundance in the resistant and susceptible wheat genotypes. Our analyses indicated that *TaRCR1* was located on chromosome 3BS of wheat. The ORF sequence of *TaRCR1* shares 87.35% and 87.07% identities with *TaRCR1*‐A and *TaRCR1*‐D, respectively. Based on the 3′‐terminal sequence of *TaRCR1*, a marker linked to sharp eyespot resistance was developed and could explain 8.00% of phenotypic variance in the recombinant inbred line (RIL) population of Shanhongmai/Wenmai6 (Table S3), which might partially explain the higher expression in the resistance lines even in the absence of infection. Several QTLs have been identified (Cai *et al*., 2006; Chen *et al*., 2013). The chromosomes 3BS harbours one QTL *QSe.cau‐3BS* represented by the marker *gwm77* in the Luke/AQ RIL population (Chen *et al*., 2013). As the marker *gwm77* on 3BS did not detect polymorphism between Shanhongmai and Wenmai6, it is impossible to analyze any link present between *TaRCR1* and *QSe.cau‐3BS* using Shanhongmai/Wenmai6 RIL population.

The functional analysis showed that *TaRCR1* overexpression significantly increased resistance of the transgenic wheat to *R. cerealis*, while *TaRCR1* silencing significantly compromised resistance to the necrotrophic fungal pathogen *R. cerealis*. The wheat line CI12633 is not immunity to the pathogen*. R. cerealis* virulent isolates could infect BSMV:00‐ and BSMV:TaRCR1‐inoculated CI12633 plants, leading to lesions in these sheaths and stems, while the lesions in BSMV:00‐inoculated CI12633 were obviously smaller than in BSMV:TaRCR1‐inoculated CI12633. These data indicated that TaRCR1, acting as a positive regulator, was required for wheat defence response to *R. cerealis*. The majority of the reported NB‐LRR proteins positively contribute to plant immunity to diverse biotrophic pathogens. In more recent documents, three distinct NB‐LRR proteins have been implicated in host susceptibility to necrotrophic fungal pathogens (Faris *et al*., 2010; Lorang *et al*., 2007; Nagy and Bennetzen, 2008). Here, to our knowledge, this study is the first to uncover the positive regulation of an NB‐LRR protein in plant resistance responses to the necrotrophic fungal pathogens. This work undoubtedly broadens understanding of biological functions of NB‐LRRs in plant species.

In plants, activation of R proteins leads to extensive transcriptional reprogramming of defence genes (Chang *et al*., 2013; Inoue *et al*., 2013; Padmanabhan *et al*., 2013; Shen *et al*., 2007; Zhu *et al*., 2010). Different regulatory proteins may activate different kinds of genes in wheat defence response to *R. cerealis*. In this study, the expression levels of *PR2*,* chitinase2*,* PR‐1.2* and *TaPIE1* were higher in *TaRCR1*‐overexperssion lines compared with WT plants, while the expression levels of *PR2* and *chitinase2* were the lowest in *TaRCR1*‐silencing plants. These results indicate that TaRCR1 may positively modulate the expression of *PR2* and *chitinase2*, whereas the expression of *PR1* and *TaPIE1* may be regulated not only by TaRCR1, but also by other proteins. The exact reprogramming mechanisms underlying TaRCR1 remain to be elucidated.

ROS signalling plays an important role in plant defence responses to pathogens. For example, ROS signalling is involved in resistance of endochitinase gene transgenic cotton to *R. solani* (Kumar *et al*., 2009). In this study, *TaRCR1* was highly induced at early stage (30 min and 3 h) of H_2_O_2_ stimulus. Furthermore, the induced transcriptional abundance of *TaRCR1* by *R. cerealis* infection was significantly elevated following by H_2_O_2_ pretreatment, suggesting that the response of *TaRCR1* to *R. cerealis* infection was associated with ROS signalling. ROS‐scavenging enzymes, including CAT, peroxidase and SOD, and ROS‐producing enzyme NOX that is also known as respiratory burst oxidase homolog (RBOH), are crucial for maintaining the ROS homoeostasis in plant cells (Kuźniak and Skłodowska, 2004; Sharma *et al*., 2007). RBOH proteins trigger cell death which is favourable for necrotrophic pathogens’ infection, whereas an *AtrbohD AtrbohF* double mutant displayed reduced cell death after infiltration with an avirulent bacterium strain (Torres *et al*., 2002; Yoshioka *et al*., 2003). Here, TaRCR1 regulates the expression of *CAT1*,* POX2* and *NOX* genes, and peroxidase activity. Consequently, the production of ROS induced after *R. cerealis* inoculation at early stage (12 and 36 h) was less in *TaRCR1*‐overexpressing wheat plants than in WT plants. At 4 dpi with *R. cerealis*, the level of ROS accumulation was reduced. These results suggested that TaRCR1 play an important role in maintaining ROS homoeostasis in wheat under *R. cerealis* stress. Further experiments indicated that peroxidase inhibition compromised TaRCR1‐mediated resistance to *R. cerealis* and that peroxidase‐dependent ROS‐scavenging might contribute to the *TaRCR1*‐mediated resistance. Additionally, the expression of defence‐associated genes (*PR2*,* PR‐1.2*,* chitinase2* and *TaPIE1*) positively regulated by TaRCR1 was enhanced upon H_2_O_2_ treatment, but markedly reduced after NaN_3_ treatment except for *PR‐1.2*, suggesting that ROS homoeostasis also regulated the expression of these genes. NaN_3_ treatment does not influence *PR‐1.2* expression, which is perhaps regulated by SOD or CAT‐mediated ROS scavenging. These results suggested that the functional role of TaRCR1 in defence response to *R. cerealis* was correlated with the expression of these defence‐associated genes that were modulated by ROS homoeostasis.

In conclusion, the wheat NB‐LRR gene *TaRCR1* was identified to be required for defence response to *R. cerealis*. TaRCR1 can regulate the expression of ROS‐scavenging and production genes, which maintains ROS homoeostasis, subsequently modulating the expression of defence genes, finally leading to enhanced resistance to *R. cerealis*. This study provides novel insights into biological functions of NB‐LRR genes in defence responses to necrotrophic pathogens.

## Experimental procedures

### Plant and fungal materials and growth conditions

The wheat lines/cultivars used in this study were Shanhongmai, CI12633, Yangmai 158, Yangmai 16 and Wenmai 6. Yangmai 16, an important planting variety in south China, is susceptible to *R. cerealis* and selected for gene transformation.

The fungus *R. cerealis* isolates R0301 and WK207 are prevailing in wheat plants of Jiangsu province and Shandong province, respectively.

All wheat plants were grown in the field or in a glasshouse at 22 °C, 14‐h light (intensity of 300 μmol/m^2^/s) and 12 °C, 10‐h dark conditions. Seedlings at the four‐leaf stage of the WT Yangmai 16 plants were sprayed with 10 mm H_2_O_2_ and 5 mm NaN_3_ (an inhibitor of peroxidase) plus 0.1% (v/v) Tween‐20 for the indicated times. Plants sprayed with water containing 0.1% Tween‐20 were used as a control for all treatments.

### Cloning and sequence analysis of *TaRCR1*

To clone the full‐length sequence of *TaRCR1* from resistant wheat line CI12633, based on the sequence of the microarray probe TC376099, primers (TaRCR1‐3′‐F1 and TaRCR1‐3′‐F2) were designed, synthetized and used to amplify the 3′‐UTR using 3′‐RACE kit v.2.0 (TaKaRa, Japan) in CI12633 and Wenmai 6 wheat plant infected by *R. cerealis* R0301 for 7 day. Then, the ORF sequence of *TaRCR1* was amplified from cDNA of CI12633 stems. A phylogenetic tree was constructed using a neighbour‐joining method implemented with MEGA 5.0 software.

### *TaRCR1* chromosomal localization

This sequence of *TaRCR1* was aligned with the wheat cv. CS genome using the service provided by IWGSC (http://wheat-urgi.versailles.inra.fr/Seq-Repository/BLAST), and the predicted chromosomal location was obtained from this website. CS nulli‐tetrasomic and di‐telosomic lines were used to verify the chromosomal localization by gene‐specific PCR.

### Subcellular localization of TaRCR1

The coding sequence of TaRCR1 was amplified by PCR and ligated to the 3′ end of the GFP coding region without the stop codon in p35S:GFP vector, generating the GFP‐TaRCR1 fusion construct p35S:GFP‐TaRCR1. The resulting p35S:GFP‐TaRCR1 and p35S:GFP constructs were separately introduced into white onion epidermal cells and wheat protoplasts following Yoo *et al*. (2007) and Zhang *et al*. (2007). After incubation at 25 °C for 20 h, GFP signals were observed and photographed using a confocal laser scanning microscope (Zeiss LSM 700) with a Fluar 10X/0.50 M27 objective lens and SP640 filter.

### BSMV‐mediated *TaRCR1* gene silencing

To generate the BSMV:TaRCR1 recombinant construct, a 315‐bp sequence of *TaRCR1* (from 2682 to 2996 nucleotides in *TaRCR1* cDNA sequence) was subcloned in an antisense orientation into the *Nhe*I restriction site of the RNAγ of BSMV (Figure S4). At the three‐leaf stage, at least 20 plants of CI12633 were inoculated with BSMV:TaRCR1 or BSMV:00 (as a control) following Holzberg *et al*. (2002). At 10 day after virus infection, the fourth leaves were collected to monitor BSMV infection and to evaluate the transcript changes of *TaRCR1*. At 14 day after BSMV infection, each stem of the BSMV‐infected CI12633 plants was inoculated with one sterile toothpick harbouring *R. cerealis* WK207 mycelia. They were scored at 14 and 21 dpi with *R. cerealis*, respectively.

### *TaRCR1* overexpression transformation vector and wheat transformation

The full ORF sequence of the *TaRCR1* gene was subcloned into modified pAHC25 vector (Christensen and Quail, 1996) with a c‐myc epitope tag, resulting in the transformation vector pUbi:myc‐TaRCR1. The vector contains the Ubi:myc‐TaRCR1‐Tnos chimera, in which c‐myc‐*TaRCR1* was driven by the maize *ubiquitin* (*Ubi*) promoter and terminated by the nopaline synthase gene (*Tnos*).

Plasmid pUBI:myc‐TaRCR1 was introduced into 2,000 immature embryos of the wheat cv. Yangmai 16 by biolistic bombardment according to the protocol described by Chen *et al*. (2008).

### PCR and Western blot analyses on *TaRCR1‐*overexpressing transgenic wheat

The presence of the introduced *TaRCR1* gene in the transformed wheat plants was monitored by PCR using a primer pair specific to the Ubi:myc‐TaRCR1‐Tnos chimera, TaRCR1‐F located in *TaRCR1* gene region and TNOS‐R located in the Tnos sequence region of the transformation vector. PCR was performed in a 20‐μL volume containing 1 μL genomic DNA (200 ng/μL), 10 μL 2 × PCR Mixture (TaKaRa), 0.5 μL each primer (10 μm) and 8 μL double distilled water.

According to Zhu *et al*. (2015), Western blots were incubated with 100‐fold diluted anti‐c‐myc antibody and secondary antibody conjugated to horseradish peroxidase (TIANGEN).

### RT‐PCR and qRT‐PCR

The expression patterns of BSMV *CP*,* TaRCR1*, ROS*‐* and defence‐related genes were analyzed by RT‐PCR or qRT‐PCR. The qRT‐PCR was operated on an ABI 7500 RT‐PCR system (Applied Biosystems, USA) following Dong *et al*. (2010). The relative expression of the target genes was calculated using the 2^−ΔΔCT^ method (Livak and Schmittgen, 2001), where the wheat *Actin* gene, *TaActin*, was used as the internal reference. Three independent biological replications were performed for each RNA sample/primer combination.

All the primers in the study are listed in Table S4.

### Assessments of *R. cerealis* responses in wheat plants

*Rhizoctonia cerealis* isolate R0301 was used to inoculate plants in T_1_ and T_2_ generations, and *R. cerealis* isolate WK207 was used to inoculate plants in T_3_ and T_4_ generations. In T_1_ and T_3_ generations in greenhouse, the *TaRCR1*‐overexpressing transgenic and WT wheat plants were grown and inoculated with *R. cerealis*. In T_2_ and T_4_ generations, the trials were conducted in nursery field. At the tillering growth stage, the plants were inoculated on each stem base with 8–10 sterile wheat kernels harbouring *R. cerealis* mycelia. To enhance humidity and *R. cerealis* infection and development, the plants were sprinkled with water twice a day during the first 7 days and then with frequency depending on rainfall and soil moisture until final disease recording. Ten to thirty plants for each line were assessed for disease severity. ITs and disease index of wheat plants were scored at ~50 dpi following Cai *et al*. (2006). At 14 dpi with *R. cerealis* WK207, the base sheaths of plants in T_4_ generation were sampled and stained by trypan blue to obverse *R. cerealis* hyphae colonization according to Peterhansel *et al*. (1997).

### Assay of ROS level and peroxidase activity

Detection of H_2_O_2_ and $O_{2}^{-}$ via histochemical staining by DAB and NBT, respectively, was performed as described by Lee *et al*. (2002). Peroxidase activity was measured using Peroxidase Activity Assay Kit (Nanjing Jiancheng Bioengineering Institute) based on the manufacturer's instruction.

## Supporting information


**Figure S1** The FoldChange of *TaRCR1* transcriptional level derived from microarray analysis (GEO accession number GSE69245) between the *R. cerealis*‐resistant wheat line CI12633/Shanhongmai and susceptible wheat cultivar Wenmai 6 at 4 and 21 days postinoculation (dpi) with *R. cerealis*.
**Figure S2** Deduced amino acid sequence of the wheat (*Triticum aestivum*) CC‐NB‐LRR gene *TaRCR1*. The conserved motifs including EDVID, P‐loop, RNBS‐A, Walker B, RNBS‐B, GLPL, RNBS‐D, and MHD were indicated in yellow.
**Figure S3** Alignment of 3′ terminal sequences of *TaRCR1* in resistant wheat line CI12633 and its homolog in susceptible wheat cultivar Wenmai 6. The software DANMAN was used to perform the sequence alignment.
**Figure S4** Scheme of genomic RNAs of the barley stripe mosaic virus (BSMV) construct and the construct of the recombinant virus expressing the wheat (*Triticum aestivum*) NB‐LRR gene *TaRCR1*, BSMV:TaRCR1. The orientation of the *TaRCR1* insert is indicated by dark boxes.
**Figure S5** qRT‐PCR analysis of *TaRCR1* in the mock (buffer‐inoculated) and BSMV:00 infected CI12633 plants. Total RNA was extracted from sheaths of mock or plants post‐BSMV:00 inoculation for 10 day. The expression level of *TaRCR1* in the mock plants was set to 1.
**Figure S6** Transcription analysis of four defence genes in the wild type (WT) wheat (*Triticum aestivum*) Yangmai 16 plants after *Rhizoctonia cerealis* inoculation for 7 day. Total RNA was extracted from sheaths of WT plants after *R. cerealis* inoculation for 7 day. The expression levels of those genes in the WT plants under normal conditions (mock treated with sterile toothpicks without pathogen) were set to 1. Significant differences between *R. cerealis* inoculation and normal conditions were derived from the results of three independent replications (*t*‐test: **, *P* < 0.01). Error bars indicate SE.
**Figure S7** Detection of hydrogen peroxide (H_2_O_2_) and superoxide anion ($O_{2}^{-}$) in wheat. Sheaths were harvested from *TaRCR1*‐overexpressing lines (R1, R12, R27), WT and *TaRCR1*‐silencing plants (BSMV:TaRCR1) and BSMV:00‐infected controls at 4 day postinfection with *R. cerealis*, and were then stained with 3,3ʹ‐diaminobenzidine (DAB) and nitroblue tetrazolium (NBT), respectively. Similar results were obtained from three independent replicates.
**Figure S8** Transcription analysis of four defence genes in the wheat (*Triticum aestivum*) Yangmai 16 plants after H_2_O_2_ and NaN_3_ treatment. Total RNA was extracted from leaves of wheat plants after H_2_O, H_2_O_2_ and NaN_3_ treatments for 12 h. The expression levels of those genes in the wheat plants treated with H_2_O were set to 1. Significant differences between H_2_O_2_ or NaN_3_ and H_2_O treatments were derived from the results of three independent replications (*t*‐test: **, *P* < 0.01). Error bars indicate SE.
**Table S1** The identities between TaRCR1 and other NLR proteins.
**Table S2** The FoldChange of *Chitinase2* and *TaPIE1* transcriptional level derived from microarray analysis (GEO accession number GSE69245).
**Table S3** *TaRCR1* conferring resistance to sharp eyespot in the Shanhongmai/Wenmai 6 RIL population.
**Table S4** Primers used in this study.

## References

[pbi12665-bib-0001] Anderson, P.A. , Lawrence, G.J. , Morrish, B.C. , Ayliffe, M.A. , Finnegan, E.J. and Ellis, J.G. (1997) Inactivation of the flax rust resistance gene *M* associated with loss of a repeated unit within the leucine‐rich repeat coding region. Plant Cell, 9, 641–651. 9144966 10.1105/tpc.9.4.641 PMC156945

[pbi12665-bib-0002] Bonardi, V. , Tang, S. , Stallmann, A. , Roberts, M. , Cherkis, K. and Dangl, J.L. (2011) Expanded functions for a family of plant intracellular immune receptors beyond specific recognition of pathogen effectors. Proc. Natl Acad. Sci. USA, 108, 16463–16468. 21911370 10.1073/pnas.1113726108 PMC3182704

[pbi12665-bib-0003] Boys, E.F. , Roques, S.E. , West, J.S. , Werner, C.P. , King, G.J. , Dyer, P.S. and Fitt, B.D.L. (2012) Effects of *R* gene‐mediated resistance in *Brassica napus* (oilseed rape) on asexual and sexual sporulation of *Pyrenopeziza brassicae* (light leaf spot). Plant. Pathol. 61, 543–554.

[pbi12665-bib-0004] Bozkurt, T.O. , Schornack, S. , Banfield, M.J. and Kamoun, S. (2012) Oomycetes, effectors, and all that jazz. Curr. Opin. Plant Biol. 15, 483–492. 22483402 10.1016/j.pbi.2012.03.008

[pbi12665-bib-0005] Cai, S. , Ren, L. , Yan, W. , Wu, J. , Chen, H. , Wu, X. and Zhang, X. (2006) Germplasm development and mapping of resistance to sharp eyespot (*Rhizoctonia cerealis*) in wheat. Sci. Agric. Sin. 39, 928–934. [In Chinese with English abstract].

[pbi12665-bib-0006] Césari, S. , Kanzaki, H. , Fujiwara, T. , Bernoux, M. , Chalvon, V. , Kawano, Y. , Shimamoto, K. *et al* (2014) The NB‐LRR proteins RGA4 and RGA5 interact functionally and physically to confer disease resistance. EMBO J. 33, 1941–1959. 25024433 10.15252/embj.201487923 PMC4195788

[pbi12665-bib-0007] Chang, C. , Yu, D. , Jiao, J. , Jing, S. , Schulze‐Lefert, P. and Shen, Q.H. (2013) Barley MLA immune receptors directly interfere with antagonistically acting transcription factors to initiate disease resistance signaling. Plant Cell, 25, 1158–1173. 23532068 10.1105/tpc.113.109942 PMC3634683

[pbi12665-bib-0008] Chen, L. , Zhang, Z. , Liang, H. , Liu, H. , Du, L. , Xu, H. and Xin, Z. (2008) Overexpression of *TiERF1* enhances resistance to sharp eyespot in transgenic wheat. J. Exp. Bot. 59, 4195–4204. 18953072 10.1093/jxb/ern259 PMC2639029

[pbi12665-bib-0009] Chen, J. , Li, G.H. , Du, Z.Y. , Quan, W. , Zhang, H.Y. , Che, M.Z. , Wang, Z. *et al* (2013) Mapping of QTL conferring resistance to sharp eyespot (*Rhizoctonia cerealis*) in bread wheat at the adult plant growth stage. Theo. Appl. Genet. 126, 2865–2878. 10.1007/s00122-013-2178-6 23989648

[pbi12665-bib-0010] Christensen, A.H. and Quail, P.H. (1996) Ubiquitin promoter‐based vectors for high‐level expression of selectable and/or screenable marker genes in monocotyledonous plants. Transgenic Res. 5, 213–218. 8673150 10.1007/BF01969712

[pbi12665-bib-0011] Cloutier, S. , McCallum, B.D. , Loutre, C. , Banks, T.W. , Wicker, T. , Feuillet, C. , Keller, B. *et al* (2007) Leaf rust resistance gene Lr1, isolated from bread wheat (*Triticum aestivum* L.) is a member of the large psr567 gene family. Plant Mol. Biol. 65, 93–106. 17611798 10.1007/s11103-007-9201-8

[pbi12665-bib-0012] Deslandes, L. , Pileur, F. , Liaubet, L. , Camut, S. , Can, C. , Williams, K. , Holub, E. *et al* (1998) Genetic characterization of *RRS1*, a recessive locus in *Arabidopsis thaliana* that confers resistance to the bacterial soilborne pathogen *Ralstonia solanacearum* . Mol. Plant Microbe Interact. 11, 659–667. 9650298 10.1094/MPMI.1998.11.7.659

[pbi12665-bib-0013] Dong, N. , Liu, X. , Lu, Y. , Du, L.P. , Xu, H.J. , Liu, H.X. , Xin, Z.Y. *et al* (2010) Overexpression of *TaPIEP1*, a pathogen‐induced ERF gene of wheat, confers host‐enhanced resistance to fungal pathogen *Bipolaris sorokiniana* . Funct. Integr. Genomics, 10, 215–226. 20225092 10.1007/s10142-009-0157-4

[pbi12665-bib-0014] Ellis, J.G. , Lawrence, G.J. , Luck, J.E. and Dodds, P.N. (1999) Identification of regions in alleles of the flax rust resistance gene *L* that determine differences in gene‐for‐gene specificity. Plant Cell, 11, 495–506. 10072407 10.1105/tpc.11.3.495 PMC144189

[pbi12665-bib-0015] Ellis, J. , Dodds, P. and Pryor, T. (2000) Structure, function and evolution of plant disease resistance genes. Curr. Opin. Plant Biol. 3, 278–284. 10873844 10.1016/s1369-5266(00)00080-7

[pbi12665-bib-0016] Faris, J.D. , Zhang, Z. , Lu, H. , Lu, S. , Reddy, L. , Cloutier, S. , Fellers, J.P. *et al* (2010) A unique wheat disease resistance‐like gene governs effector‐triggered susceptibility to necrotrophic pathogens. Proc. Natl Acad. Sci. USA, 107, 13544–13549. 20624958 10.1073/pnas.1004090107 PMC2922177

[pbi12665-bib-0017] Feuillet, C. , Travella, S. , Stein, N. , Albar, L. , Nublat, A. and Keller, B. (2003) Map‐based isolation of the leaf rust disease resistance gene *Lr10* from the hexaploid wheat (*Triticum aestivum* L.) genome. Proc. Natl Acad. Sci. USA, 79, 893–903. 10.1073/pnas.2435133100 PMC299976 14645721

[pbi12665-bib-0018] Foley, R.C. , Kidd, B.N. , Hane, J.K. , Anderson, J.P. and Singh, K.B. (2016) Reactive Oxygen Species Play a Role in the Infection of the Necrotrophic Fungi, *Rhizoctonia solani* in Wheat. PLoS ONE, 11, e0152548. 27031952 10.1371/journal.pone.0152548 PMC4816451

[pbi12665-bib-0019] Gabriëls, S.H. , Vossen, J.H. , Ekengren, S.K. , van Ooijen, G. , Abd‐El‐Haliem, A.M. , van den Berg, G.C. , Rainey, D.Y. *et al* (2007) An NB‐LRR protein required for HR signalling mediated by both extra‐ and intracellular resistance proteins. Plant J. 50, 14–28. 17346268 10.1111/j.1365-313X.2007.03027.x

[pbi12665-bib-0020] Garcia‐Brugger, A. , Lamotte, O. , Vandelle, E. , Bourque, S. , Lecourieux, D. , Poinssot, B. , Wendehenne, D. *et al* (2006) Early signaling events induced by elicitors of plant defenses. Mol. Plant Microbe Interact. 19, 711–724. 16838784 10.1094/MPMI-19-0711

[pbi12665-bib-0021] Heller, J. and Tudzynski, P. (2011) Reactive oxygen species in phytopathogenic fungi: signaling, development, and disease. Annu. Rev. Phytopathol. 49, 369–390. 21568704 10.1146/annurev-phyto-072910-095355

[pbi12665-bib-0022] Hinsch, M. and Staskawicz, B. (1996) Identification of a new Arabidopsis disease resistance locus, *RPs4*, and cloning of the corresponding avirulence gene, *avrRps4*, from *Pseudomonas syringae* pv. *pisi* . Mol. Plant Microbe Interact. 9, 55–61. 8589423 10.1094/mpmi-9-0055

[pbi12665-bib-0023] Holzberg, S. , Brosio, P. , Gross, C. and Pogue, G.P. (2002) Barley stripe mosaic virus‐induced gene silencing in a monocot plant. Plant J. 30, 315–327. 12000679 10.1046/j.1365-313x.2002.01291.x

[pbi12665-bib-0024] Huang, L. , Brooks, S.A. , Li, W. , Fellers, J.P. , Trick, H.N. and Gill, B.S. (2003) Map‐based cloning of leaf rust resistance gene *Lr21* from the large and polyploid genome of bread wheat. Genetics, 164, 655–664. 12807786 10.1093/genetics/164.2.655 PMC1462593

[pbi12665-bib-0025] Huang, X.Q. , Hsam, S.L. , Mohler, V. , Röder, M.S. and Zeller, F.J. (2004) Genetic mapping of three alleles at the *Pm3* locus conferring powdery mildew resistance in common wheat (*Triticum aestivum* L.). Genome, 47, 1130–1136. 15644971 10.1139/g04-079

[pbi12665-bib-0026] Inoue, H. , Hayashi, N. , Matsushita, A. , Xinqiong, L. , Nakayama, A. , Sugano, S. , Jiang, C.J. *et al* (2013) Blast resistance of CC‐NB‐LRR protein Pb1 is mediated by WRKY45 through protein‐protein interaction. Proc. Natl Acad. Sci. USA, 110, 9577–9582. 23696671 10.1073/pnas.1222155110 PMC3677490

[pbi12665-bib-0027] Jones, J.D. and Dangl, J.L. (2006) The plant immune system. Nature, 444, 323–329. 17108957 10.1038/nature05286

[pbi12665-bib-0028] Kumar, V. , Parkhi, V. , Kenerley, C. and Rathore, K. (2009) Defense‐related gene expression and enzyme activities in transgenic cotton plants expressing an endochitinase gene from *T*richoderma virens in response to interaction with *Rhizoctonia solani* . Planta, 230, 277–291. 19444464 10.1007/s00425-009-0937-z

[pbi12665-bib-0029] Kuźniak, E. and Skłodowska, M. (2004) The effect of *Botrytis cinerea* infection on the antioxidant profile of mitochondria from tomato leaves. J. Exp. Bot. 55, 605–612. 14966215 10.1093/jxb/erh076

[pbi12665-bib-0030] Lee, B.H. , Lee, H. , Xiong, L. and Zhu, J.K. (2002) A mitochondrial complex I defect impairs cold‐regulated nuclear gene expression. Plant Cell, 14, 1235–1251. 12084824 10.1105/tpc.010433 PMC150777

[pbi12665-bib-0031] Livak, K.J. and Schmittgen, T.D. (2001) Analysis of relative gene expression data using real‐time quantitative PCR and the 2^−▵▵CT^ Method. Methods, 25, 402–408. 11846609 10.1006/meth.2001.1262

[pbi12665-bib-0032] Lorang, J.M. , Sweat, T.A. and Wolpert, T.J. (2007) Plant disease susceptibility conferred by a “resistance” gene. Proc. Natl Acad. Sci. USA, 104, 14861–14866. 17804803 10.1073/pnas.0702572104 PMC1976202

[pbi12665-bib-0033] Mittler, R. (2002) Oxidative stress, antioxidants and stress tolerance. Trends Plant Sci. 7, 405–410. 12234732 10.1016/s1360-1385(02)02312-9

[pbi12665-bib-0034] Nagy, E.D. and Bennetzen, J.L. (2008) Pathogen corruption and site‐directed recombination at a plant disease resistance gene cluster. Genome Res. 18, 1918–1923. 18719093 10.1101/gr.078766.108 PMC2593579

[pbi12665-bib-0035] Padmanabhan, M.S. , Ma, S. , Burch‐Smith, T.M. , Czymmek, K. , Huijser, P. and Dinesh‐Kumar, S.P. (2013) Novel positive regulatory role for the SPL6 transcription factor in the N TIR‐NB‐LRR receptor‐mediated plant innate immunity. PLoS Pathog. 9, e1003235. 23516366 10.1371/journal.ppat.1003235 PMC3597514

[pbi12665-bib-0036] Pan, Y. , Bradley, G. , Pyke, K. , Ball, G. , Lu, C. , Fray, R. , Marshall, A. *et al* (2013) Network Inference Analysis Identifies an APRR2‐Like Gene Linked to Pigment Accumulation in Tomato and Pepper Fruits. Plant Physiol. 161, 1476–1485. 23292788 10.1104/pp.112.212654 PMC3585610

[pbi12665-bib-0037] Periyannan, S. , Moore, J. , Ayliffe, M. , Bansal, U. , Wang, X. , Huang, L. , Deal, K. *et al* (2013) The gene *Sr33*, an ortholog of barley *Mla* genes, encodes resistance to wheat stem rust race Ug99. Science, 341, 786–788. 23811228 10.1126/science.1239028

[pbi12665-bib-0038] Peterhansel, C. , Freialdenhoven, A. , Kurth, J. , Kolsch, R. and Schulze, L.P. (1997) Interaction analyses of genes required for resistance responses to powdery mildew in barley reveal distinct pathways leading to leaf cell death. Plant Cell, 9, 1397–1409. 12237388 10.1105/tpc.9.8.1397 PMC157006

[pbi12665-bib-0039] Quan, L.J. , Zhang, B. , Shi, W.W. and Li, H.Y. (2008) Hydrogen peroxide in plants: a versatile molecule of the reactive oxygen species network. J. Integr. Plant Biol. 50, 2–18. 18666947 10.1111/j.1744-7909.2007.00599.x

[pbi12665-bib-0040] Saintenac, C. , Zhang, W. , Salcedo, A. , Rouse, M.N. , Trick, H.N. , Akhunov, E. and Dubcovsky, J. (2013) Identification of wheat gene *Sr35* that confers resistance to Ug99 stem rust race group. Science, 341, 783–786. 23811222 10.1126/science.1239022 PMC4748951

[pbi12665-bib-0041] Sharma, N. , Rahman, M.H. , Strelkov, S. , Thiagarajah, M. , Bansal, V.K. and Kav, N.N. (2007) Proteome‐level changes in two *Brassica napus* lines exhibiting differential responses to the fungal pathogen *Alternaria brassicae* . Plant Sci. 172, 95–110.

[pbi12665-bib-0042] Shen, Q.H. , Saijo, Y. , Mauch, S. , Biskup, C. , Bieri, S. , Keller, B. , Seki, H. *et al* (2007) Nuclear activity of MLA immune receptors links isolate‐specific and basal disease‐resistance responses. Science, 315, 1098–1103. 17185563 10.1126/science.1136372

[pbi12665-bib-0043] Shetty, N.P. , Jøgersen, H.J.L. , Jensen, J.D. , Collinge, D.B. and Shetty, H.S. (2008) Roles of reactive oxygen species in interactions between plants and pathogens. Eur. J. Plant Pathol. 121, 267–280.

[pbi12665-bib-0044] Spies, N. , Burge, C.B. and Bartel, D.P. (2013) 3′ UTR‐isoform choice has limited influence on the stability and translational efficiency of most mRNAs in mouse fibroblasts. Genome Res. 23, 2078–2090. 24072873 10.1101/gr.156919.113 PMC3847777

[pbi12665-bib-0045] Stotz, H.U. , Mitrousia, G.K. , de Wit, P.J.G.M. and Fitt, B.D.L. (2014) Effector‐triggered defence against apoplastic fungal pathogens. Trends Plant Sci. 19, 491–500. 24856287 10.1016/j.tplants.2014.04.009 PMC4123193

[pbi12665-bib-0046] Thirugnanasambandam, A. , Wright, K.M. , Atkins, S.D. , Whisson, S.C. and Newton, A.C. (2011) Infection of *Rrs1* barley by an incompatible race of the fungus *Rhynchosporium secalis* expressing the green fluorescent protein. Plant. Pathol. 60, 513–521.

[pbi12665-bib-0047] Torres, M.A. , Dangl, J.L. and Jones, J.D.G. (2002) Arabidopsis gp91phox homologues *AtrbohD* and *AtrbohF* are required for accumulation of reactive oxygen intermediates in the plant defense response. Proc. Natl Acad. Sci. USA, 99, 517–522. 11756663 10.1073/pnas.012452499 PMC117592

[pbi12665-bib-0048] Valent, B. and Chang, H.K. (2010) Recent advances in rice blast effector research. Curr. Opin. Plant Biol. 13, 434–441. 20627803 10.1016/j.pbi.2010.04.012

[pbi12665-bib-0049] Wei, X. , Shen, F. , Hong, Y. , Rong, W. , Du, L. , Liu, X. , Xu, H. *et al* (2016) The wheat calcium‐dependent protein kinase TaDPK7‐D positively regulates host resistance to sharp eyespot disease. Mol. Plant Pathol. 17, 1252–1264. 26720854 10.1111/mpp.12360 PMC6638438

[pbi12665-bib-0050] Whitham, S. , Dinesh‐Kumar, S.P. , Choi, D. , Hehl, R. , Corr, C. and Baker, B. (1994) The product of the tobacco mosaic virus resistance gene *N*: similarity to toll and the interleukin‐1 receptor. Cell, 78, 1101–1115. 7923359 10.1016/0092-8674(94)90283-6

[pbi12665-bib-0051] Wu, L. , Zhang, Z. , Zhang, H. , Wang, X.C. and Huang, R. (2008) Transcriptional modulation of ethylene response factor protein JERF3 in the oxidative stress response enhances tolerance of tobacco seedlings to salt, drought, and freezing. Plant Physiol. 148, 1953–1963. 18945933 10.1104/pp.108.126813 PMC2593663

[pbi12665-bib-0052] Wu, C.H. , Belhaj, K. , Bozkurt, T.O. , Birk, M.S. and Kamoun, S. (2016) Helper NLR proteins NRC2a/b and NRC3 but not NRC1 are required for Pto‐mediated cell death and resistance in *Nicotiana benthamiana* . New Phytol. 209, 1344–1352. 26592988 10.1111/nph.13764

[pbi12665-bib-0053] Yoo, S.D. , Cho, Y.H. and Sheen, J. (2007) Arabidopsis mesophyll protoplasts: a versatile cell system for transient gene expression analysis. Nature Protoc. 2, 1565–1572. 17585298 10.1038/nprot.2007.199

[pbi12665-bib-0054] Yoshioka, H. , Numata, N. , Nakajima, K. , Katou, S. , Kawakita, K. , Rowland, O. and Doke, N. (2003) *Nicotiana benthamiana* gp91^phox^ homologs *NbrbohA* and *NbrbohB* participate in H_2_O_2_ accumulation and resistance to *Phytophthora infestans* . Plant Cell, 15, 706–718. 12615943 10.1105/tpc.008680 PMC150024

[pbi12665-bib-0055] Zhan, W.B. , Chen, J. , Zhang, Z.D. , Zhou, L. and Fukuda, H. (2003) Elimination of shrimp endogenous peroxidase background in immunodot blot assays to detect white spot syndrome virus (WSSV). Dis. Aquat. Organ. 53, 263–265. 12691198 10.3354/dao053263

[pbi12665-bib-0056] Zhang, Z. , Yao, W. , Dong, N. , Liang, H. , Liu, H. and Huang, R. (2007) A novel ERF transcription activator in wheat and its induction kinetics after pathogen and hormone treatments. J. Exp. Bot. 58, 2993–3003. 17728298 10.1093/jxb/erm151

[pbi12665-bib-0057] Zhu, Z. , Xu, F. , Zhang, Y. , Cheng, Y.T. , Wiermer, M. , Li, X. and Zhang, Y. (2010) *Arabidopsis* resistance protein SNC1 activates immune responses through association with a transcriptional corepressor. Proc. Natl Acad. Sci. USA, 107, 13960–13965. 20647385 10.1073/pnas.1002828107 PMC2922275

[pbi12665-bib-0058] Zhu, X. , Qi, L. , Liu, X. , Cai, S. , Xu, H. , Huang, R. , Li, J. *et al* (2014) The wheat ethylene response factor transcription factor PATHOGEN‐INDUCED ERF1 mediates host responses to both the necrotrophic pathogen *Rhizoctonia cerealis* and freezing stresses. Plant Physiol. 164, 1499–1514. 24424323 10.1104/pp.113.229575 PMC3938636

[pbi12665-bib-0059] Zhu, X. , Yang, K. , Wei, X. , Zhang, Q. , Rong, W. , Du, L. , Ye, X. *et al* (2015) The wheat AGC kinase TaAGC1 is a positive contributor to host resistance to the necrotrophic pathogen *Rhizoctonia cerealis* . J. Exp. Bot. 66, 6591–6603. 26220083 10.1093/jxb/erv367 PMC4623678

