# Supplementary material for: The wheat NB‐LRR gene TaRCR1 is required for host defence response to the necrotrophic fungal pathogen Rhizoctonia cerealis
Source: Plant Biotechnol J. 2017 Mar 1;15(6):674–87. doi: 10.1111/pbi.12665 (PMC5425395; doi:10.1111/pbi.12665)
Supplement: Supplementary file 1 — Figure S1 The FoldChange of TaRCR1 transcriptional level derived from microarray analysis (GEO accession number GSE69245) between the R. cerealis‐resistant wheat line CI12633/Shanhongmai and susceptible wheat cultivar Wenmai 6 at 4 and 21 days postinoculation (dpi) with R. cerealis. Figure S2 Deduced amino acid sequence of the wheat (Triticum aestivum) CC‐NB‐LRR gene TaRCR1. The conserved motifs including EDVID, P‐loop, RNBS‐A, Walker B, RNBS‐B, GLPL, RNBS‐D, and MHD were indicated in yellow. Figure S3 Alignment of 3′ terminal sequences of TaRCR1 in resistant wheat line CI12633 and its homolog in susceptible wheat cultivar Wenmai 6. The software DANMAN was used to perform the sequence alignment. Figure S4 Scheme of genomic RNAs of the barley stripe mosaic virus (BSMV) construct and the construct of the recombinant virus expressing the wheat (Triticum aestivum) NB‐LRR gene TaRCR1, BSMV:TaRCR1. The orientation of the TaRCR1 insert is indicated by dark boxes. Figure S5 qRT‐PCR analysis of TaRCR1 in the mock (buffer‐inoculated) and BSMV:00 infected CI12633 plants. Total RNA was extracted from sheaths of mock or plants post‐BSMV:00 inoculation for 10 day. The expression level of TaRCR1 in the mock plants was set to 1. Figure S6 Transcription analysis of four defence genes in the wild type (WT) wheat (Triticum aestivum) Yangmai 16 plants after Rhizoctonia cerealis inoculation for 7 day. Total RNA was extracted from sheaths of WT plants after R. cerealis inoculation for 7 day. The expression levels of those genes in the WT plants under normal conditions (mock treated with sterile toothpicks without pathogen) were set to 1. Significant differences between R. cerealis inoculation and normal conditions were derived from the results of three independent replications (t‐test: **, P < 0.01). Error bars indicate SE. Figure S7 Detection of hydrogen peroxide (H2O2) and superoxide anion (O2−) in wheat. Sheaths were harvested from TaRCR1‐overexpressing lines (R1, R12, R27), WT and Ta [file PBI-15-674-s001.doc]

**Supporting Information**

**The wheat NB-LRR gene *TaRCR1* is required for host defence response to the necrotrophic fungal pathogen *Rhizoctonia cerealis***

Xiuliang Zhu, Chungui Lu, Lipu Du, Xingguo Ye, Xin Liu, Anne Coules, Zengyan Zhang^*^

**Supplementary Figures**

**Figure S1.** The FoldChange of *TaRCR1* transcriptional level derived from microarray analysis (GEO accession number GSE69245) between the *R. cerealis*-resistant wheat line CI12633/Shanhongmai and susceptible wheat cultivar Wenmai 6 at 4 and 21 days post inoculation (dpi) with *R. cerealis*.

**
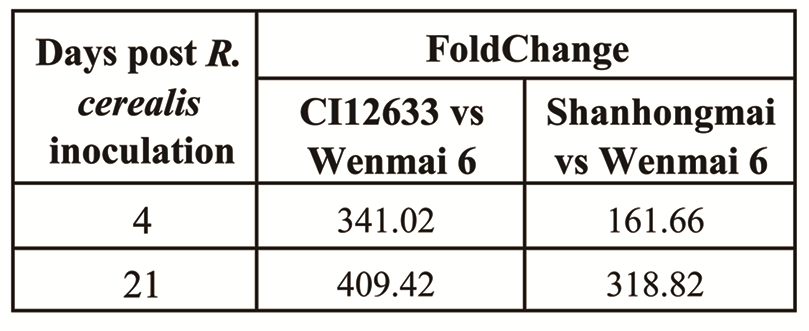
**

**Figure S2.** Deduced amino acid sequence of the wheat (*Triticum aestivum*) CC-NB-LRR gene *TaRCR1*. The conserved motifs including EDVID, P-loop, RNBS-A, Walker B, RNBS-B, GLPL, RNBS-D, and MHD were indicated in yellow.


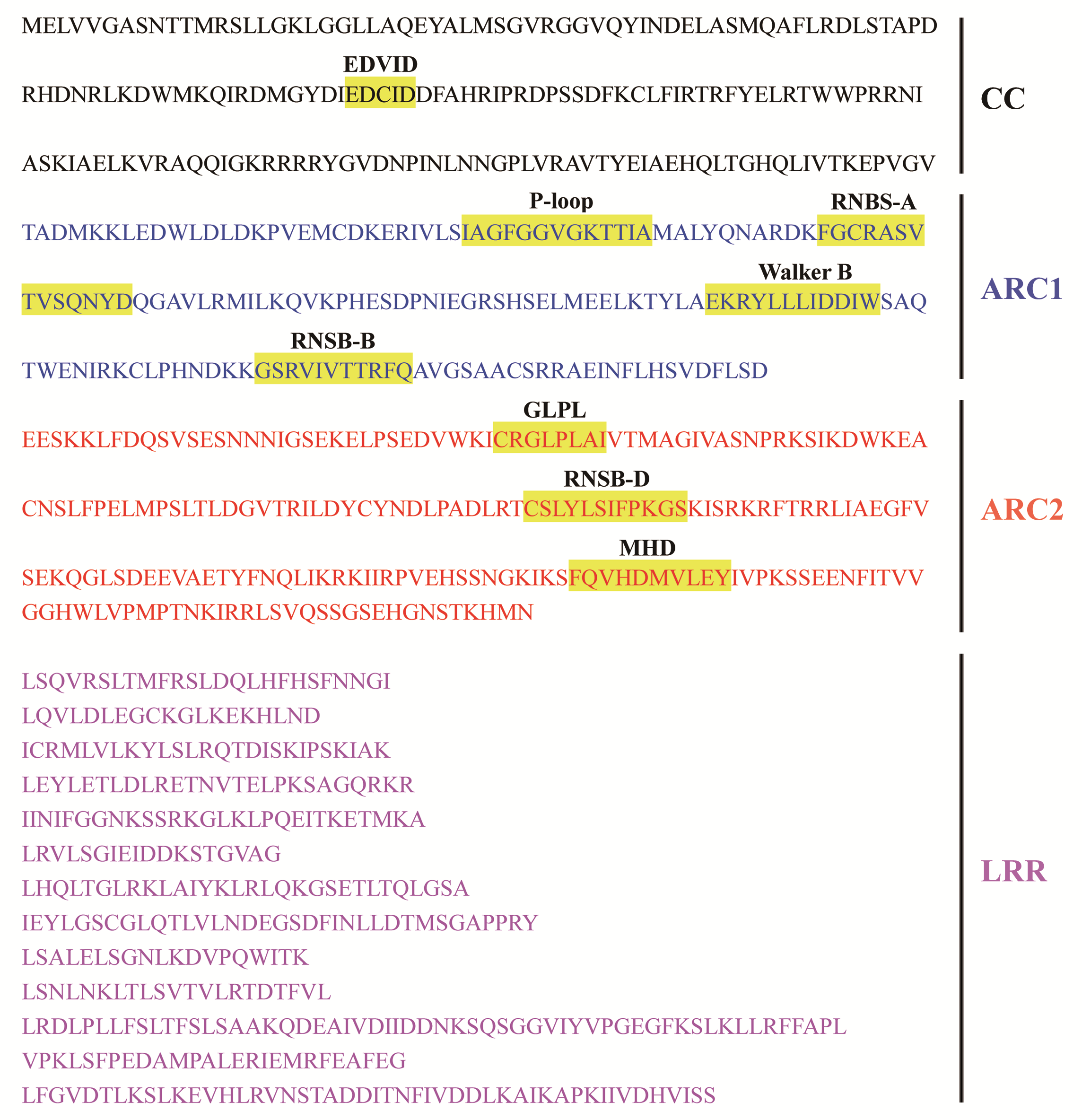


**Figure S3.** Alignment of 3′ terminal sequences of *TaRCR1* in resistant wheat line CI12633 and its homolog in susceptible wheat cultivar Wenmai 6. The software DANMAN was used to perform the sequence alignment.


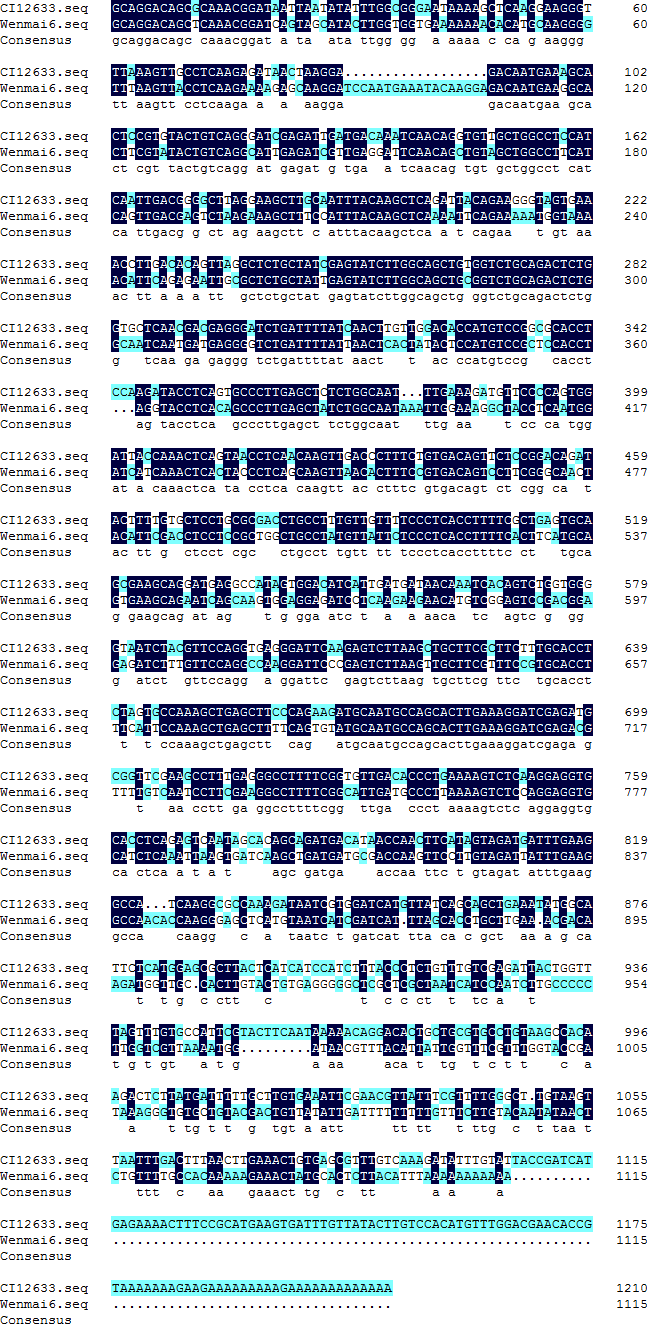


**Figure S4.** Scheme of genomic RNAs of the barley stripe mosaic virus (BSMV) construct and the construct of the recombinant virus expressing the wheat (*Triticum aestivum*) NB-LRR gene *TaRCR1*, BSMV:TaRCR1. The orientation of the *TaRCR1* insert is indicated by dark boxes.

**
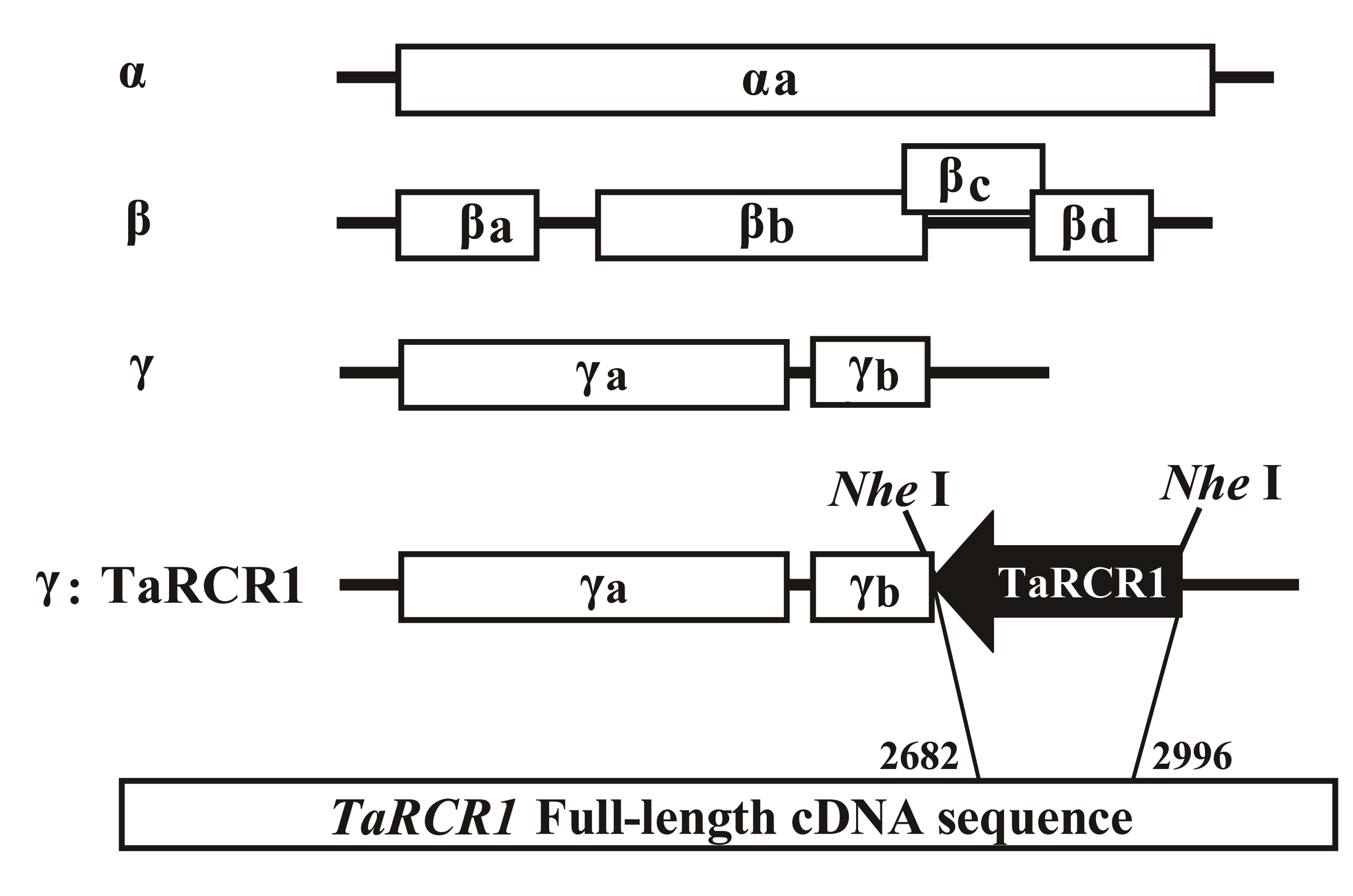
**

**Figure S5.** qRT-PCR analysis of *TaRCR1* in the mock (buffer-inoculated) and BSMV:00 infected CI12633 plants. Total RNA was extracted from sheaths of mock or plants post BSMV:00 inoculation for 10 d. The expression level of *TaRCR1* in the mock plants was set to 1.


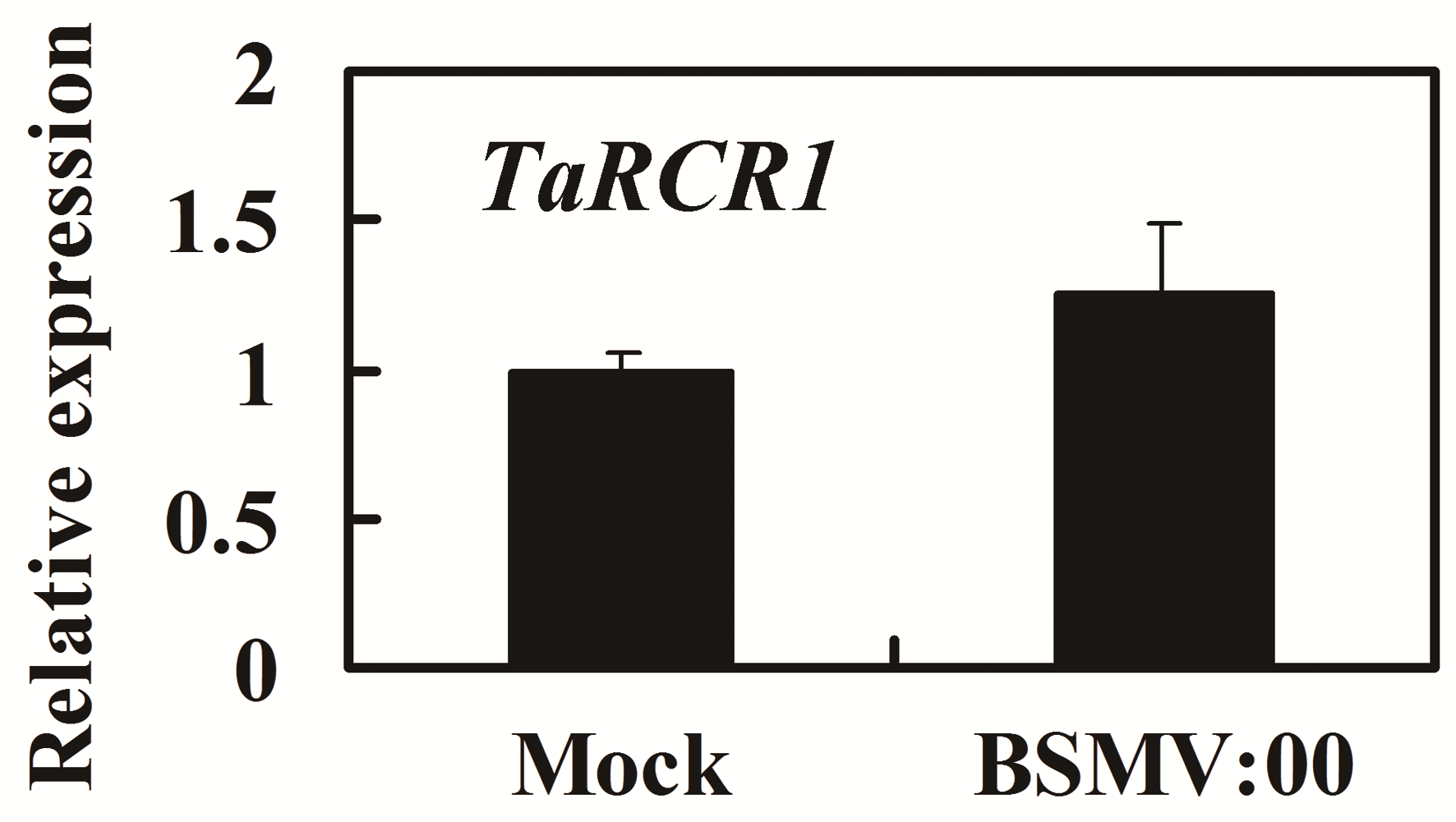


**Figure S6.** Transcription analysis of four defence genes in the wild type (WT) wheat (*Triticum aestivum*) Yangmai 16 plants after *Rhizoctonia cerealis* inoculation for 7 d. Total RNA was extracted from sheaths of WT plants after *R. cerealis* inoculation for 7 d. The expression levels of those genes in the WT plants under normal conditions (mock treated with sterile toothpicks without pathogen) were set to 1. Significant differences between *R. cerealis* inoculation and normal conditions were derived from the results of three independent replications (*t*-test: **, P < 0.01). Error bars indicate SE.

**
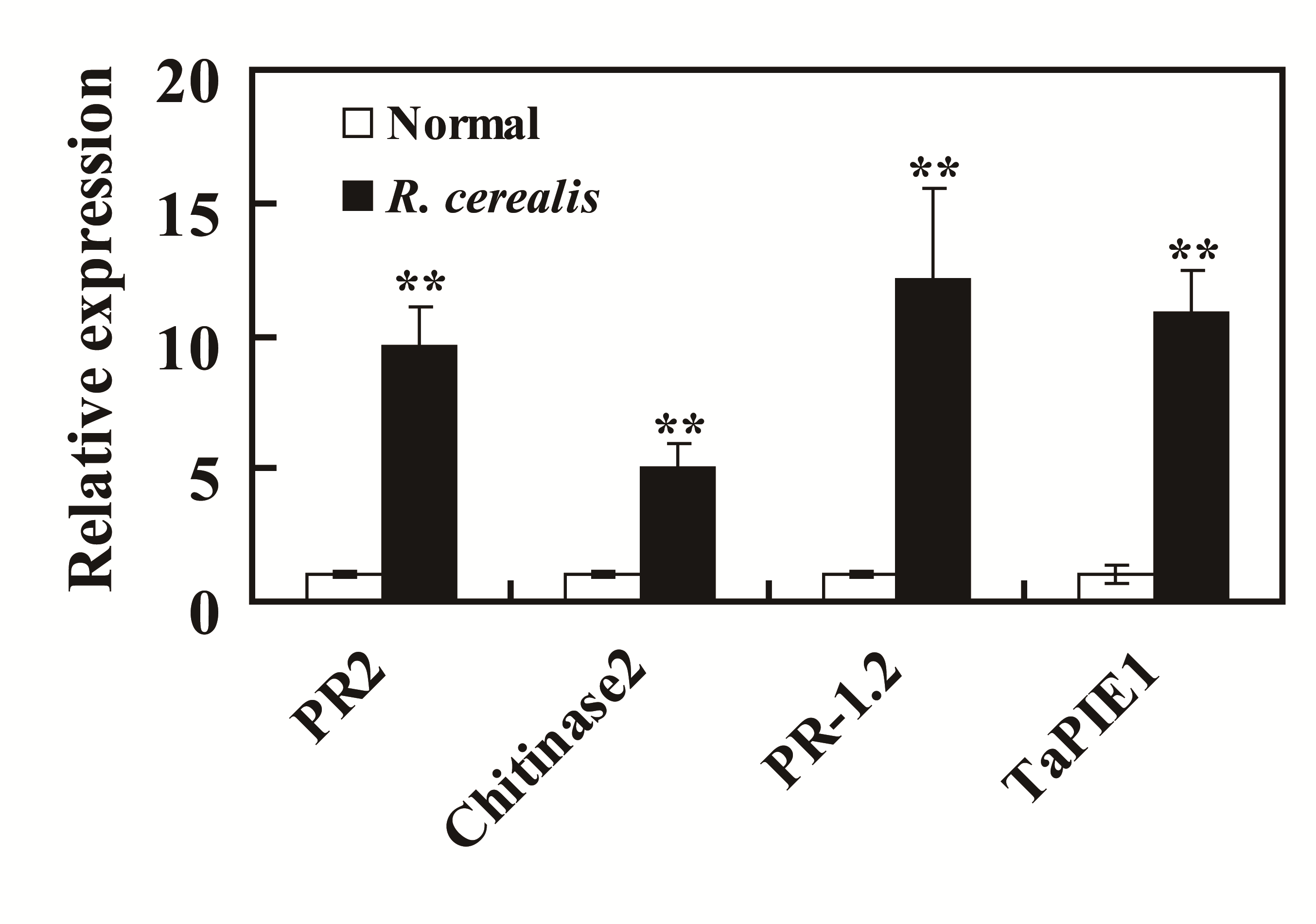
**

**Figure S7.** Detection of hydrogen peroxide (H_2_O_2_) and superoxide anion (O_2_^−^) in wheat. Sheaths were harvested from *TaRCR1*-overexpressing lines (R1, R12, R27), WT and *TaRCR1*-silencing plants (BSMV:TaRCR1) and BSMV:00-infected controls at 4 d post infection with *R. cerealis*, and were then stained with 3,3ʹ-diaminobenzidine (DAB) and nitroblue tetrazolium (NBT), respectively. Similar results were obtained from three independent replicates.

**
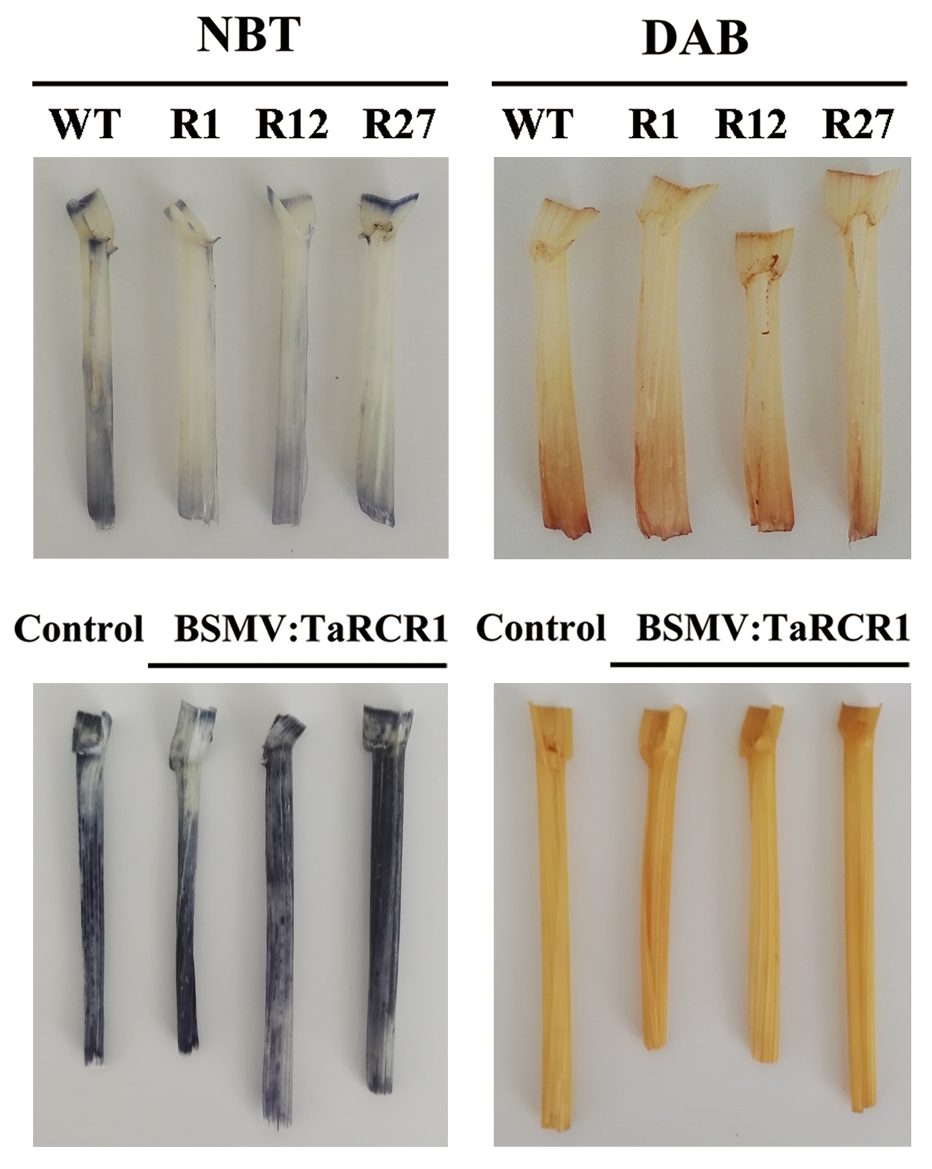
**

**Figure S8.** Transcription analysis of four defence genes in the wheat (*Triticum aestivum*) Yangmai 16 plants after H_2_O_2_ and NaN_3_ treatment. Total RNA was extracted from leaves of wheat plants after H_2_O, H_2_O_2_ and NaN_3_ treatments for 12 h. The expression levels of those genes in the wheat plants treated with H_2_O were set to 1. Significant differences between H_2_O_2_ or NaN_3_ and H_2_O treatments were derived from the results of three independent replications (*t*-test: **, P < 0.01). Error bars indicate SE.


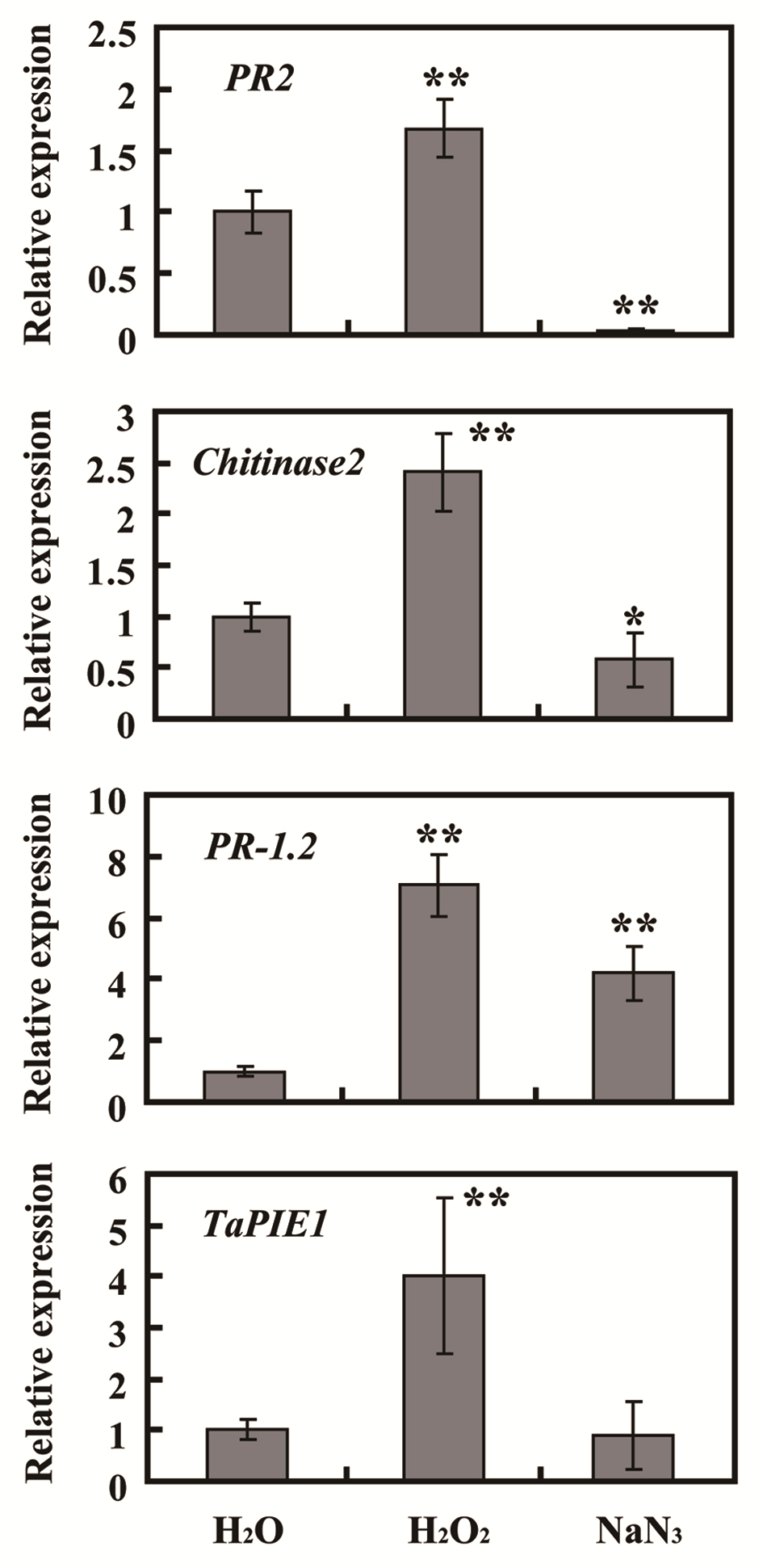


**Supplementary Tables**

**Table S1.** The identities between TaRCR1 and other NLR proteins

| Species | Protein | GenBank accession number | Identity (%) |
| --- | --- | --- | --- |
| *Oryza sativa* | Yr10 | BAM28949.1 | 61.28 |
|  | Xa1 | BAA25068.1 | 7.05 |
| *Aegilops tauschii* | Lr21 | AAP74647.1 | 10.80 |
| *Triticum aestivum* | Lr1 | ABS29034.1 | 9.11 |
|  | Lr10 | AAQ01784.1 | 23.39 |
|  | TaRCR1-A | KX840356 | 82.45 |
|  | TaRCR1-D | KX840357 | 81.38 |
|  | Sr33 | AGQ17390.1 | 23.78 |
| *Triticum monococcum* | Sr35 | AGP75918.1 | 26.12 |
|  | TmMla1 | ADX06722.1 | 24.67 |
| *Hordeum vulgare* | HvMla1 | AAG37356.1 | 25.03 |
|  | HvMla7 | AAQ55540.1 | 24.82 |
|  | HvMla10 | AAQ55541.1 | 25.57 |
| *Zea mays* | Rp1-D | AAD47197.1 | 9.86 |
| *Solanum lycopersicum* | NRC1 | ABC26878.1 | 18.69 |
| *Arabidopsis thaliana* | RLM | Q9FT77.1 | 9.62 |

**Table S2.** The FoldChange of *Chitinase2* and *TaPIE1* transcriptional level derived from microarray analysis (GEO accession number GSE69245)

| **Genes** | **Days post *R. cerealis* inoculation** | **FoldChange** | |
| --- | --- | --- | --- |
|  |  | **CI12633 vs Wenmai 6** | **Shanhongmai vs Wenmai 6** |
|  |  |  |  |
| *Chitinase2* | 4 | 1.84 | 4.90 |
|  | 21 | 10.02 | 6.27 |
| *TaPIE1* | 4 | 13.12 | 0.37 |
|  | 21 | 23.52 | 2.67 |

The microarray analysis was between the *Rhizoctonia cerealis*-resistant wheat line CI12633/Shanhongmai and susceptible wheat cultivar Wenmai 6 at 4 and 21 days post inoculation with *R. cerealis*.

**Table S3.** *TaRCR1* conferring resistance to sharp eyespot in the Shanhongmai/ Wenmai 6 RILpopulation

| Marker | PVE (%) | LOD |
| --- | --- | --- |
| RCR1 | 8.0046 | 1.9002 |

**Table S4.** Primers used in this study

| Primer name | Accession number | Sequence of  gene-specific primer | Usage |
| --- | --- | --- | --- |
| TaRCR1-3′-F1 | KU161103 | 5′-CTTGACCTAAGGGAGACGAA-3′ | 3′ RACE for *TaRCR1* |
| TaRCR1-3′-F2 |  | 5′- GCAGGACAGCTCAAACGGA-3′ |  |
| TaRCR1-ORF-F1 |  | 5′- TCCACAGAACGGGCAAAC-3′ | Amplification of full-length cDNA of *TaRCR1* |
| TaRCR1-ORF-R1 |  | 5′- CAAGCCCAAAACGAAATAAC-3′ |  |
| TaRCR1-ORF-F2 |  | 5′- CCCAGTCAAAGTCCGTCAGT-3′ |  |
| TaRCR1-ORF-R2 |  | 5′-GTGGCTTACAGGCACGCAG-3′ |  |
| TaRCR1-2950F |  | 5′- GCACCTCAGAGTCAATAGCAC-3′ | Detection of transformed *TaRCR1* |
| TaRCR1-3214R |  | 5′- AAAACCCATCTCATAAATAACG-3′ |  |
| TaRCR1-Q-F |  | 5′- ACTAAGGAGACAATGAAAGCAC -3′ | qRT-PCR for *TaRCR1* transcript |
| TaRCR1-Q-R |  | 5′- CCCTCGTCGTTGAGCACC -3′ |  |
| TaRCR1-P25-F |  | 5′-AACTCGGTATCTAGAATGGAGTTGGTAGTAGGTGC-3′ | Construction of vector pUBI: myc-TaRCR1 |
| TaRCR1-P25-R |  | 5′-CGATCGGGGAAATTCTCAGCTGCTGATAACATGAT-3′ |  |
| TaRCR1-γ-F |  | 5′- TACGCTAGCGCGACCTGCCTTTGTTGT-3′ | Construction of vector γ-TaRCR1 |
| TaRCR1-γ-R |  | 5′- GACGCTAGCTGTCATCTGCTGTGCTATTG-3′ |  |
| TaRCR1-GFP-F |  | 5′-ATGGACGAGCTGTACAAGATGGAGTTGGTAGTAGGTGC-3′ | Construction of vector pGFP-TaRCR1 |
| TaRCR1-GFP-R |  | 5′-TCAGCGTACCGAATTCCCTCAGCTGCTGATAACATGAT-3′ |  |
| TaRCR1-A-F1 | KX840356 | 5′- ACTTGATTCACGAAGGTAGA-3′ | Amplification of full-length cDNA of *TaRCR1-A* |
| TaRCR1-A-R1 |  | 5′- AATAGGAGTCTTGCGGTGT-3′ |  |
| TaRCR1-A-F2 |  | 5′- TTCCATAAAGATATCGGGAT-3′ |  |
| TaRCR1-A-R2 |  | 5′- AGAAAAGTGCTCCAATAGAATG-3′ |  |
| TaRCR1-D-F1 | KX840357 | 5′- CAGTTGCTAAGGTTCTGTAAAA-3′ | Amplification of full-length cDNA of *TaRCR1-D* |
| TaRCR1-D-R1 |  | 5′- AAGCCCAGAATGAAATAAGGT -3′ |  |
| TaRCR1-D-F1 |  | 5′- GATTTGGATACTGAAGAACACA-3′ |  |
| TaRCR1-D-R1 |  | 5′- TAATCTCAACAAACAGAGTGGA -3′ |  |
| BSMV-CP-F | JF803284 | 5′-TGACTGCTAAGGGTGGAGGA-3′ | Detection of BSMV virus |
| BSMV-CP-R |  | 5′-CGGTTGAACATCACGAAGAGT-3′ |  |
| RcActin-F | KJ631110 | 5′- gcatccacgagaccacttac-3′ | Detection of *R. cerealis* |
| RcActin-R |  | 5′- gcgtcccgctgctcaagat-3′ |  |
| TaActin-F | BE425627 | 5′-CACTGGAATGGTCAAGGCTG-3′ | Internal control for qRT-PCR |
| TaActin-R |  | 5′-CTCCATGTCATCCCAGTTG-3′ |  |
| POX2-QF | X85228 | 5′- AGGGGCTTCGGCGTCATC-3′ | qRT-PCR for *POX2* transcript |
| POX2-QR |  | 5′- TTGGGCGTCGTCGTGTCC-3′ |  |
| TaCAT1-QF | GU984379 | 5′-CAAGGGCTTCTTCGAGGTCAC-3′ | qRT-PCR for *TaCAT1* transcript |
| TaCAT1-QR |  | 5′- TGTAGAAGGTCCACTCCGGGTAG-3′ |  |
| TaNOX-QF | AY561153 | 5′-ATGTTCGGCAACTTGGTGACT-3′ | qRT-PCR for *TaNOX* transcript |
| TaNOX-QR |  | 5′- CGTCTGCTCTAAGAAGACCACTTTT-3′ |  |
| PR1.2-QF | AJ007349 | 5′- CGTCTTCATCACCTGCAACTA-3′ | qRT-PCR for *PR1.2* transcript |
| PR1.2-QR |  | 5′- CAAACATAAACACACGCACGTA-3′ |  |
| PR2-QF | AF112965 | 5′- CCGCACAAGACACCTCAAGATA-3′ | qRT-PCR for *PR2* transcript |
| PR2-QR |  | 5′- CGATGCCCTTGGTTTGGTAGA-3′ |  |
| TaPIE1-QF | EF583940 | 5′- GGAGCCACCAGTCCGTATGA-3′ | qRT-PCR for *TaPIE1* transcript |
| TaPIE1-QR |  | 5′- CACCCGGCAGAGGTATTCAA-3′ |  |
| Chitinase2-QF | TC426538 | 5′-TTCTGGATGACGGCACAAG-3′ | qRT-PCR for *Chitinase2* transcript |
| Chitinase2-QR |  | 5′-CCTTAGTGTGACCAGTCGTTTT-3′ |  |
